# Environmental Pollution and Related Redox Pathways Contributing to Retinal Diseases

**DOI:** 10.3390/antiox15101258

**Published:** 2026-10-01

**Authors:** Liyu Zhang, Qi Tang, Elsa Wilma Böhm, Felix Mathias Wagner, Adrian Gericke

**Affiliations:** 1 Department of Ophthalmology, University Medical Center of the Johannes Gutenberg University Mainz, Langenbeckstr. 1, 55131 Mainz, Germany; qitang.med@gmail.com (Q.T.); elsawilma.boehm@unimedizin-mainz.de (E.W.B.); felix.mathias.wagner@gmail.com (F.M.W.); 2 Vista Alpina Eye Clinic, Bahnhofplatz 1A, 3930 Visp, Switzerland

**Keywords:** environment, pollution, eye, pathophysiology, retina, diseases

## Abstract

Environmental pollution, a detrimental consequence of industrialization and globalization, is increasingly recognized as a potential risk factor for a wide range of human diseases, including retinal disorders. The retina is particularly vulnerable to environmental stressors, including particulate matter, nitrogen oxides, carbon monoxide, traffic-related noise, and extreme environmental conditions such as elevated temperatures. Epidemiological and experimental evidence suggests that these exposures may contribute to the development and progression of retinal diseases through mechanisms involving oxidative stress, chronic inflammation, immune dysregulation, and vascular dysfunction. These processes may ultimately contribute to retinal vasculopathies and degenerative disorders. Consequently, the pathophysiological mechanisms linking environmental exposures to retinal injury have received increasing attention in recent years. This review provides a disease-centered perspective on the relationship between environmental pollution and retinal diseases, integrating epidemiological evidence with experimental findings across specific retinal disorders. Among the environmental pollutants investigated to date, fine particulate matter (PM_2.5_) and nitrogen dioxide (NO_2_) have the most consistent epidemiological evidence for adverse effects on retinal health, particularly in relation to age-related macular degeneration (AMD). The review also discusses the methodological limitations and heterogeneity of the available evidence, as well as current knowledge gaps and future research perspectives.

## 1. Introduction

Environmental pollution, including air pollution, traffic noise, ambient heat, chemical contamination, and light pollution, has emerged as a major public health concern worldwide [1]. According to the World Health Organization (WHO), around 4.2 million deaths occur annually as a result of the pathological effects of air pollution [2]. Ambient air pollution has steadily increased over the past two decades, especially in cities [3]. Pollutants are released as byproducts of industrial activities (e.g., mining, manufacturing, construction) [4], motor vehicle fuel combustion, household energy use, and natural events such as forest fires, dust storms, and pollen dispersion. These pollutants include gases such as nitrogen dioxide (NO_2_), sulfur dioxide (SO_2_), ozone (O_3_), and carbon monoxide (CO), as well as particulate matter (PM) [5]. Moreover, transportation noise has been identified as a potential cardiovascular risk factor in recent years, which, according to the WHO, is associated with the loss of around 1.6 million healthy life-years in Europe annually [6].

Although the relationship between environmental pollutants, respiratory, neurological, and cardiovascular diseases is well-established, the connection between environmental pollution and retinal diseases remains comparatively underexplored [7,8,9,10,11,12,13,14,15,16,17,18,19].

Retinal diseases, including age-related macular degeneration (AMD) and diabetic retinopathy (DR), are leading causes of severe visual impairment and blindness in adults [20]. Children are also affected by visual disorders, such as inherited retinal diseases (IRD) and retinopathy of prematurity (ROP) [21]. A global assessment of vision impairment in 2020 revealed that an estimated 43.3 million people were blind; 295 million suffered from moderate to severe vision impairment; and 258 million had mild vision impairment. Projections suggest that by 2050, these numbers will rise to 61 million, 474 million, and 360 million, respectively [22]. Furthermore, the estimated global annual economic productivity loss associated with vision impairment was $411 billion in 2020 [23].

As one of the most metabolically active tissues in the human body, the retina, supports the substantial energy demands of photoreceptor transduction. Its exceptionally high oxygen consumption and electron transport chain activity place it in a naturally high-risk state for oxidative stress, including ROS generated through mitochondrial electron leakage [24]. Additionally, both the retina and retinal pigment epithelium (RPE) are enriched with long-chain polyunsaturated fatty acids (PUFAs), which are highly susceptible to ROS-mediated damage. This vulnerability promotes lipid peroxidation and the formation of toxic byproducts such as 4-hydroxynonenal (4-HNE) and lipid peroxides, further contributing to cellular injury [25]. These physiological characteristics render the retina highly susceptible to oxidative injury from environmental or endogenous stressors, making it a primary target for degenerative eye diseases such as AMD and DR.

While other recent review articles are focused on molecular mechanisms by which environmental pollutants induce oxidative stress, inflammation, mitochondrial dysfunction, and cell death in ocular tissues, the present article aims to provide a disease-centered perspective of the emerging link between environmental pollution and retinal diseases by integrating epidemiological evidence and experimental findings to specific retinal diseases.

The literature was identified via search on PubMed. The following keywords were used: (“environmental pollution” OR “particulate matter” OR “carbon monoxide” OR “noise” OR “toxins” OR “light pollution” OR “nanoplastics”) AND (“oxidative stress” OR “inflammation” OR “endothelial function” OR “retinal diseases” OR “age-related macular degeneration” OR “diabetic retinopathy” OR “retinal vein occlusion” OR “central artery occlusion” OR “retinal detachment” OR “retinoblastoma” OR “retinopathy of prematurity” OR “retinal dysplasia” OR “congenital pigmentary retinopathy”). The research was performed from 12 December 2025 to 10 March 2026. The following inclusion criteria were determined: all studies, written in English, and published after 1990. The reference list of the selected articles was reviewed for further identification of relevant studies.

## 2. General Molecular Pathomechanisms Triggered by Pollutants

### 2.1. Oxidative Stress

Environmental pollutants induce oxidative stress through convergent yet molecule-specific signaling cascades in various retinal cell types.

For example, particulate matter, classified as PM_2.5_ (aerodynamic diameter ≤ 2.5 μm) and PM_10_ (≤10 μm), can be inhaled and deposited in the respiratory tract, with PM_2.5_ being sufficiently small to penetrate the alveoli and enter the bloodstream [26,27,28,29]. Evidence from mouse studies indicates that PM exposure induces oxidative stress and endothelial dysfunction in both cerebral and retinal blood vessels [30]. The same study further demonstrates that co-exposure to PM and traffic noise exerts synergistic neurovascular toxicity. This combined exposure triggers oxidative stress and inflammatory signaling pathways in the brain, characterized by NADPH oxidase 2 (NOX2)-dependent overproduction of reactive oxygen species (ROS), impairment of antioxidant defenses, and nuclear factor kappa B (NF-κB)-mediated inflammatory activation. Together, these mechanisms may contribute to the elevated risk of neurodegenerative, cardiovascular, and cerebrovascular diseases in urban populations [30].

Specifically, PM_2.5_ particles were shown to induce transcriptomic reprogramming and activation of transforming growth factor-beta (TGF-β) signaling, enhancing cellular migration, as well as epithelial–mesenchymal transition via the PI3K/AKT/mTOR pathway in human retinal pigment epithelial (RPE) cells [31,32]. Moreover, PM_2.5_ promoted ROS generation and activated the stress response kinase, c-Jun NH2-terminal kinase (JNK) [33].

Ultrafine diesel exhaust particles have been shown to activate NOX2 in isolated oligodendrocytes from rat brains, causing a sharp rise in intracellular ROS and elevating the expression of tumor protein p53 (p53), Bcl-2-associated X protein (Bax), and caspase-3, ultimately inducing apoptosis [34]. Since NOX2 is also a known source of ROS within the retinal tissue that shares many characteristics with the central nervous system, the presented NOX2-driven pathophysiological mechanisms might also be relevant for retinal diseases [35].

Diesel exhaust particles have also been shown to induce inflammation and blood–retinal barrier (BRB) dysfunction through toll-like receptor 2 (TLR2) and TLR4 activation, leading to increased vascular permeability and potential retinal damage [36]. Moreover, diesel PM_2.5_ was reported to promote epithelial–mesenchymal transition of human RPE cells via generation of ROS and activation of the TGF-β/Smad/ERK/p38 MAPK signaling pathway [37].

Apart from PM, heavy metals may similarly disrupt redox homeostasis. For example, cadmium, a heavy metal environmental toxin known for its neurotoxic effects on the central nervous system, has been implicated in glaucoma and AMD. In human retinal organoids, cadmium exposure caused a dose- and time-dependent reduction in neural retinal thickness and organoid volume. Mechanistically, cadmium exposure induced cell apoptosis, inhibited retinal progenitor cell proliferation, and affected ganglion cell differentiation [38].

In RPE cells, cadmium also triggered ROS generation and activation of the MAPK pathway including JNK, ERK1/2, and p38. Antioxidants such as N-acetylcysteine (NAC) significantly reduced cadmium-induced toxicity. These results indicate that elevated ROS-induced activation of the MAPK signaling pathway could be associated with cadmium-induced RPE cell apoptosis, one of the major contributing factors in AMD [39]. Another study in RPE cells demonstrated that cadmium induced apoptosis and dose-dependent cell viability loss, and activated caspase-3. Moreover, endoplasmic reticulum stress was activated as its marker, binding immunoglobulin protein, was remarkably upregulated by cadmium exposure [40].

In rats, cadmium exposure induced inflammatory and proapoptotic responses, leading to significant histopathological damage in the retina [41].

In a study on human ocular tissue, higher cadmium levels were found in the neural retina and RPE for eyes afflicted with AMD compared to controls in males; however, differences were not statistically significant in females [42]. The results indicate that higher retinal cadmium burdens are associated with the presence of AMD at least in males and suggest possible gender differences in the metabolism of metals in the human retina.

Persistent organic pollutants and pesticides also provoke oxidative injury through mitochondrial and antioxidant-system disruption. For example, exposure to benzo[a]pyrene, a well-known procarcinogen, dose-dependently reduced cell viability and significantly increased caspase-3/7, -8, -9, and -12 activities in RPE cells [43]. Another study reported that benzo[a]pyrene caused autophagy in RPE cells, but not apoptosis. Notably, the antioxidant substances, resveratrol and quercetin, prevented benzo[a]pyrene-induced autophagy [44]. Furthermore, a study that tested the effects of benzo(e)pyrene on retinal neurosensory (R28) cells and human microvascular endothelial cells (HMVEC), found different mechanisms of action of the substance depending on the cell type. While in R28 cells, benzo(e)pyrene caused activation of caspase and apoptotic cell death at moderate concentrations, but non-apoptotic cell death at higher concentrations, in HMVEC benzo(e)pyrene induced cell death via a non-caspase-dependent necrosis pathway [45]. Benzo[a]pyrene also decreases the endothelium-dependent nitric oxide (NO)-induced vasodilation in porcine retinal arterioles through the production of superoxide from NADPH oxidase, which is linked to JNK and p38 kinase. The results suggested that endoplasmic reticulum stress is instrumental in benzo[a]pyrene-induced endothelial dysfunction and that antioxidant substances, such as genistein and resveratrol, may preserve endothelial function [46].

Figure 1 summarizes the principal molecular pathogenic mechanisms initiated by environmental pollutants that converge on oxidative stress.

### 2.2. Vasoconstriction

Recent studies have demonstrated that a wide range of environmental pollutants can directly activate vascular endothelial and smooth muscle cells, triggering both acute and chronic vasoconstriction. For example, in a double-blind, crossover, controlled-exposure study in human volunteers, diesel exhaust rapidly increased plasma endothelin-1 (ET-1) concentrations in exposed adults [47]. Acting through the ETA receptor (EDNRA), ET-1 increases Ca^2+^ influx into smooth muscle cells and promotes myosin light chain phosphorylation, leading to rapid contraction of large blood vessels as demonstrated in pulmonary arteries. With sustained exposure, endothelial nitric oxide synthase (eNOS) becomes downregulated and NO production declines, weakening the vasodilatory counterbalance to ET-1 and further intensifying contractile tension [47,48].

Chronic low-dose lead exposure in rats might induce hypertension by upregulating the vasoconstrictive peptide endothelin-3 (ET-3) in the blood plasma and decreasing plasma und urinary cGMP concentrations as a potential indicator for an impaired NO–cGMP relaxation pathway, thus shifting vascular tone towards ET-3-mediated contraction [49]. Mercury ions, in contrast, inactivate eNOS by binding to sulfur/selenium groups, enhance NOX activity and mitochondrial electron leakage, markedly increase ROS generation, and deplete NO. This combined disruption of the NO–cGMP relaxation axis is accompanied by mercury-induced increases in ET-1, angiotensin II (Ang II), and cyclooxygenase-2 (COX-2) products, which collectively amplify the ETA/AT_1_ receptor–Ca^2+^–myosin light chain kinase (MLCK) signaling cascade, which may drive strong smooth muscle contraction, rapidly elevating vascular tone [50].

Arsenite further enhances vascular reactivity by promoting Ca^2+^ sensitization through increased phosphorylation of smooth muscle myosin light chain. This intensifies vasoconstriction mediated by the α_1A_-adrenoceptor and the 5-HT_2_A (HTR2A) serotonin receptor, thereby augmenting vasoconstive responses to phenylephrine, serotonin, and elevated extracellular potassium (K^+^) in isolated aortic rings [51]. Together, these mechanisms reveal a unifying pathogenic pattern across diverse pollutant exposures: suppression of the antioxidant–NO axis combined with upregulation of vasoconstrictive peptides (ET-1, Ang II) and their receptors, culminating in a clinical phenotype of vasospasm and elevated blood pressure. Figure 2 provides an overview of the molecular signaling pathways by which pollutants induce vasoconstriction.

### 2.3. Inflammation

From fine particulate matter in vehicle exhaust to volatile organic compounds released by industrial activity, environmental pollutants silently undermine human health. Through interconnected mechanisms, including oxidative stress, organelle dysfunction, and amplification of receptor-mediated signaling, pollutants effectively ignite inflammatory cascades within the body. Direct evidence from pollutant-induced inflammatory signaling in retinal cells remains limited. However, studies in other tissue types have identified conserved inflammatory pathways, that might also be relevant to retinal pathophysiology.

As demonstrated in lung tissue, PM_2.5_ promotes the release of interleukin-1β (IL-1β) by activating wingless-type MMTV integration site family member 5A (Wnt5a)-dependent autophagy, which subsequently triggers NOD-like receptor family pyrin domain containing 3 (NLRP3) inflammasome activation [52]. Diesel exhaust particles exacerbate inflammation through a ROS–ERK–cFOS signal amplification loop that upregulates pro-inflammatory genes, including interferon-γ (IFN-γ), IL-1β, IL-6, and NLRP3, while simultaneously suppressing the anti-inflammatory cytokine IL-10 in human Wharton’s jelly-derived mesenchymal stem cells [53]. Perfluorooctanesulfonic acid or its salt, perfluorooctanesulfonate (PFOS), released via wastewater effluents, atmospheric deposition, and landfill leachate, persists in the environment due to its exceptional chemical stability and resistance to degradation [54]. PFOS accumulates in aquatic ecosystems, biomagnifies through food webs, and ultimately reaches humans primarily through contaminated drinking water and consumption of contaminated fish and seafood [55]. Mechanistically, PFOS directly activates toll-like receptor 4 (TLR4), which recruits caspase-8 to initiate NLRP3 inflammasome activation. This leads to caspase-1-dependent maturation of IL-1β, driving inflammatory injury in photoreceptor cells under PFOS exposure as found in zebrafish and photoreceptor cell models [56]. Beyond the induction of inflammatory cytokines, activation of the NLRP3 inflammasome can also trigger pyroptosis by activation of caspase-1 and promotion of IL-1β release, as demonstrated in human corneal epithelial cells after exposure to particulate matter [57]. Pyroptosis that involves formation of pores into cell membranes with subsequent cell rupture and release of cellular components has been shown to be a relevant contributor to retinal injury in retinal diseases, such as diabetic retinopathy, and can further aggravate inflammation, initiating a vicious circle of cellular damage [58,59].

Figure 3 summarizes the principal signaling pathways through which pollutants induce inflammation and activate immune responses.

### 2.4. Blood–Retinal Barrier (BRB) Dysfunction

Exposure to airborne and chemical pollutants can compromise the integrity of the blood–retinal barrier (BRB), leading to retinal edema and contributing to the development of multiple retinal disorders. Diesel exhaust particles (DEPs) and PM_2.5_ have been shown to downregulate key tight junction proteins, includingclaudin-5, zonula occludens-1 (ZO-1), and occludin—in both retinal endothelial cells and retinal pigment epithelial (RPE) cells, thereby increasing BRB permeability [36,60,61]. In vitro and in vivo studies indicate that DEP-activated macrophages suppress claudin-5 and ZO-1 expression through TLR2/4-mediated signaling [36], while PM_2.5_ exposure disrupts tight junction integrity (ZO-1, occludin) in mouse RPE cells [60]. Consistently, chronic PM_2.5_ exposure in animal models induces retinal vascular leakage and edema, reflecting inner BRB breakdown [61]. Together, these findings suggest that pollutant-induced BRB dysfunction arises primarily from tight junction disassembly and increased vascular permeability, representing a key pathogenic mechanism contributing to AMD and other retinal vascular diseases.

### 2.5. Non-Apoptotic Cell Death and Autophagy

Environmental pollutants can induce non-apoptotic cell death in retinal cells. For example, exposure to PM_2.5_ [61], cigarette smoke [62], or blue light in the presence of N-retinylidene-N-retinylethanolamine, a leading component of RPE lipofuscin [63], can provoke ferroptosis, characterized by iron-dependent lipid peroxidation in RPE or retinal microvascular cells, marked by Fe^2+^ accumulation, depletion of Glutathione (GSH), downregulation of the SLC7A11/GPX4 axis, and enhanced lipid peroxidation. These detrimental effects can be effectively mitigated by ferrostatin-1, iron chelators, or pigment epithelium-derived factor (PEDF) [64]. In contrast, autophagy is a cellular stress response that maintains cellular homeostasis through degradation of damaged proteins and organelles. Urban particulate matter has been shown to trigger autophagy in RPE cells, rather than classical apoptosis [65]. This response might initially be protective, but in case of dysregulation during sustained stress it might contribute to retinal cellular damage. Multiple studies indicate that targeting mitochondrial homeostasis and autophagic activity can help to preserve photoreceptors or RPE cells from such injuries [66].

### 2.6. Aryl Hydrocarbon Receptor (AHR) Signaling Activation and Metabolic Reprogramming

Some studies have shown that co-exposure to polycyclic aromatic hydrocarbons (PAHs), such as indenopyrene, and high-energy blue light strongly activates AHR signaling in RPE cells. This activation is accompanied by upregulation of cytochrome P450 family 1 subfamily A member 1 (CYP1A1) and cytochrome P450 family 1 subfamily B member 1 (CYP1B1), leading to excessive Phase I metabolic activity and suppression of Phase II detoxification. As a result, ROS and DNA adducts accumulate, supporting a mechanism referred to as “metabolic phase uncoupling” [67].

Under physiological conditions, the AHR regulates immune responses and maintains barrier homeostasis in the outer retina and choroid [68]. Loss or functional impairment of AHR induces a pro-inflammatory phenotype in RPE cells, characterized by increased expression of intercellular adhesion molecule-1 (ICAM-1) [68]. Acting as an environmental sensor, AHR also modulates lipid and glucose metabolism and influences mitochondrial function through the cytochrome P450 (CYP) enzyme system and redox-regulatory pathways, thereby contributing to the metabolic dysregulation observed in AMD and DR [69]. These apparently opposing findings highlight the context-dependent role of AHR signaling. Since physiological AHR signaling is essential for retinal homeostasis and immune regulation, persistent activation or dysregulation induced by environmental pollutants can have harmful effects promoting oxidative stress and inflammation [68].

## 3. Environmental Pollution and Age-Related Macular Degeneration (AMD)

### 3.1. General Aspects of AMD

AMD is a leading cause of central vision loss, particularly in individuals over 50 years old. According to the WHO, AMD accounts for 8.7% of global blindness [70]. With population aging, its prevalence continues to rise, and by 2040 an estimated 288 million people worldwide are expected to be affected [71]. The economic burden is substantial. Annual costs attributable to late-stage AMD are estimated at $512.5 million in Bulgaria, $8.6 billion in Germany, and $49.4 billion in the United States, with direct medical expenses accounting for 10–13% of total expenditures across countries [72]. AMD is a multifactorial degenerative disease of the central retina characterized by progressive loss of choroidal capillaries, retinal pigment epithelium, and photoreceptors. Clinically, these structural changes manifest as central blurring, metamorphopsia (perception of distorted lines), and a relative central scotoma [73,74]. Despite therapeutic advances, long-term outcomes remain limited. A 2025 real-world 10-year meta-analysis involving more than 7500 eyes found that although anti-vascular endothelial growth factor (VEGF) therapy yielded early visual improvements in neovascular AMD, mean visual acuity still declined by approximately eight letters over a decade, highlighting the need for more durable and optimized treatment strategies [75]. The pathophysiology of AMD is multifactorial, involving the interplay of genetic susceptibility and environmental risk factors [76,77]. Figure 4 shows a clinical picture of neovascular AMD.

### 3.2. Air Pollution and AMD

#### 3.2.1. Epidemiological Association Between Air Pollution and AMD

A longitudinal, population-based study using data from the Taiwan National Health Insurance Program between 1998 and 2010 investigated the effects of long-term exposure to environmental NO_2_ and CO on AMD risk. The study supports evidence that exposure to high levels of NO_2_ and CO significantly increased the risk of AMD, with hazard ratios (HRs) of 1.91 and 1.84, respectively, for individuals in the highest quartile of exposure [78,79]. Similarly, a meta-analysis evaluated the epidemiological evidence regarding the associations between exposure to multiple air pollutants and the risk of AMD [80]. Chang et al. reported a positive association between higher exposure to CO and NO_2_ and AMD risk [78]. Consistent with these findings, a cross-sectional analysis of participants from the UK Biobank demonstrated that residence in areas with higher PM_2.5_ concentrations was associated with a greater prevalence of self-reported AMD. This association was further supported by optical coherence tomography (OCT) findings, which revealed structural alterations in retinal layers among individuals exposed to higher levels of PM_2.5_, PM_10_, NO_2_, and nitric oxides (NO_x_). However, whether these OCT-detected changes are specifically related to AMD pathogenesis or instead reflect more general pollution-induced retinal toxicity remains unclear [81]. Additional evidence was provided by an analysis of the Canadian Longitudinal Study on Aging, a multicenter observational study in which annual mean pollutant concentrations were estimated based on participants’ postal codes. Higher residential exposure to PM_2.5_ was associated with an increased risk of visually impairing AMD. Notably, this association was not observed in individuals with early-stage AMD, suggesting that PM_2.5_ exposure may have a greater impact on advanced disease, particularly the late neovascular stage associated with substantial visual impairment [82]. However, the association between PM_2.5_ and AMD was no longer statistically significant in multipollutant models [82]. These epidemiological findings should therefore be interpreted with caution, as the reported exposure estimates were derived from residential land-use regression models rather than individual long-term exposure measurements. Consequently, estimated exposure may not accurately reflect individual cumulative exposure, and residual confounding by other environmental, socioeconomic, or lifestyle factors cannot be excluded. In rural China, researchers evaluated the combined effects of meteorological factors and PM_2.5_ on AMD, suggesting a positive correlation between AMD risk and levels of PM_2.5_, temperature, and relative humidity. These findings provide evidence that environmental interactions extend beyond air pollution alone [83]. A nationwide cross-sectional study in China reported associations of O_3_ exposure with increased AMD risk, particularly during warmer months when O_3_ concentrations exceeded 110 μg/m^3^ [84].

Smoking represents another important environmental risk factor with well-established adverse effects on systemic and ocular health. In the EUREYE study, a population-based investigation of early age-related maculopathy and AMD among individuals aged ≥65 years from seven European countries, current smoking was associated with an increased risk of neovascular AMD (OR 2.6), while former smokers also exhibited an elevated risk (OR, approximately 1.7). Bilateral disease was more frequently observed among individuals with a history of smoking, suggesting a potential cumulative association between tobacco exposure and AMD development and progression [85].

An increased susceptibility to AMD was also reported among passive smokers in the The Blue Mountains Eye Study, a cross-sectional, population-based study involving more than 3000 participants in Australia. Consistent with findings for other environmental pollutants, the association between smoking exposure and AMD appeared to be stronger for late-stage disease than for early AMD [86]. However, this apparent difference should be interpreted cautiously, as it may reflect greater cumulative lifetime exposure or longer disease duration rather than a stage-specific effect of smoking. Moreover, the analysis was adjusted only for age and sex, leaving the possibility of residual confounding by other established AMD risk factors.

#### 3.2.2. Pathophysiological Impact of Air Pollutants in AMD

Several studies have demonstrated that cigarette smoke extract induces oxidative damage and apoptosis in RPE cells, contributing to AMD by disrupting mitochondrial membrane potential, increasing superoxide production, and depleting intracellular GSH levels [87]. In addition, a study investigating the effects of environmental tobacco smoke on microRNA (miRNA) expression in Rhesus macaques revealed significant alterations in miRNA profiles in both the circulatory system and in the vitreous. This suggests that tobacco smoke exposure may play a role in AMD pathogenesis by modulating miRNA expression [88]. Perfluorinated compounds, particularly perfluorooctanoic acid (PFOA), found in indoor dust, have also been linked to AMD risk factors [89]. Other studies have proposed that ambient air pollutants, including PM_2.5_, NO_2_, and SO_2_, may increase AMD risk by promoting oxidative stress, chronic inflammation, and lipid deposition, key mechanisms underlying both wet (neovascular) and dry (geographic atrophy) forms of advanced AMD [90]. The retina is particularly susceptible to oxidative damage triggered by environmental pollutants. Age-related oxidative stress further amplifies this vulnerability, leading to retinal dysfunction and RPE cell loss. Air pollutants such as PM, O_3_, NO_2_, and SO_2_ can penetrate the alveolar walls, enter the bloodstream, cross the BRB, and reach retinal cells, where they may contribute to AMD development [91,92]. Higher levels of PM_2.5_ and nitrogen oxides have been associated with significant structural retinal alterations, such as thinner retinal nerve fiber layer, inner nuclear layer, outer plexiform layer, and outer nuclear layer [93]. Furthermore, dust pollution caused oxidative stress and disrupted antioxidant defense mechanisms in cell culture studies of retinal Muller glia cells, thereby potentially contributing to retinal dysfunction [94].

Taken together, environmental pollution influences AMD development through multiple molecular pathways, including the regulation of metabolic enzymes, oxidative stress, and inflammation. These findings underscore the importance of reducing exposure to air pollution and smoking as preventive strategies, while also identifying potential targets for future therapeutic interventions.

### 3.3. Light Pollution and AMD

#### 3.3.1. Epidemiological Association Between Light Pollution and AMD

Degenerative retinal diseases such as dry and neovascular AMD, retinitis pigmentosa, and DR are characterized by progressive degeneration of the RPE. Damage to this layer ultimately leads to photoreceptor cell death, resulting in partial or complete vision loss. A national population-based case–control study conducted in Korea investigated the relationship between outdoor artificial nighttime lighting (OALAN) and the risk of exudative AMD in 126,418 participants [95]. The study concluded that higher levels of residential outdoor artificial light at night were associated with a higher risk of developing exudative AMD, particularly in urban areas [95]. However, further studies incorporating individual-level exposure assessments and potential mediators, including other environmental factors, are warranted, particularly in urban areas where light pollution is more prevalent. Blue light corresponds to the shorter-wavelength, higher-energy portion of the visible light spectrum. Although sunlight remains the predominant environmental source of blue light, modern electronic devices, including smartphones, laptops, and tablets, contribute substantially to daily blue light exposure [96]. Unlike ultraviolet (UV) radiation, blue light penetrates ocular tissues more efficiently and is encountered frequently in daily life [97].

To establish safety thresholds for ocular exposure to optical radiation, the International Commission on Non-Ionising Radiation Protection (ICNIRP) has published detailed exposure guidelines [98]. Importantly, blue light emissions from contemporary lighting and display technologies remain substantially below the ocular exposure limits defined by the ICNIRP [99].

#### 3.3.2. Pathophysiological Impact of Light Pollution in AMD

Because of its high oxygen consumption, the retina is particularly vulnerable to oxidative stress [100,101]. Blue light-induced cell death is mediated by apoptotic processes involving caspase-3 [102,103,104] and p53 activation [105]. Mitochondrial damage triggered by blue light, along with the resulting burst of ROS, is recognized as a major contributor to the degeneration of retinal cells, including those of the RPE [106,107]. Additionally, blue light exposure has been linked to ferroptosis, a regulated form of cell death driven by imbalance in the GSH-GPX4 and FSP1-CoQ10-NADH systems, which plays a key role in retinal pathology [108].

Observational clinical studies have examined the effectiveness of blue light-filtering intraocular lenses (BF-IOLs), designed to reduce short-wavelength visible light as a potential strategy to protect the retina and prevent AMD. While some reports have suggested a possible protective effect of BF-IOLs on AMD onset and progression, the overall evidence remains inconsistent [109,110]. Other studies, including a nationwide cohort study from Taiwan with a mean follow-up of 6.1 years, found no significant difference in AMD incidence or progression between patients receiving BF-IOLs and those implanted with non-BF-IOLs [111,112,113]. A Cochrane systematic review encompassing 51 randomized controlled trials comparing BF- and non-BF-IOLs in adults undergoing cataract surgery similarly concluded that there is no evidence that BF-IOLs preserve macular health or influence AMD development or progression [114,115]. Another systematic review by Vagge et al. reached the same conclusion, finding insufficient evidence to support the clinical use of BF-IOLs for retinal protection [116].

In summary, although laboratory studies clearly demonstrate that blue light promotes retinal degeneration through caspase-3/p53-mediated apoptosis, mitochondrial ROS generation and ferroptosis pathways, real-world clinical evidence does not currently support the protective effectiveness of blue light-filtering IOLs. These discrepancies underscore the need for well-designed, long-term clinical trials to clarify the potential benefits of BF-IOLs.

### 3.4. Nanoplastic Exposure and AMD

The widespread use of plastics has resulted in significant environmental, social, economic, and health consequences. Over time, plastics degrade into nanoplastics (NPs), defined by a diameter of less than 1 micron, through the effects of sunlight, wind, heat, and physical abrasion. NPs can enter the human body via inhalation, ingestion, and skin contact, and have been detected in human excretions as well as in nearly every organ examined. Growing research attention has focused on the biological effects of NPs on living organisms, such as the impact of long-term exposure to polystyrene nanoplastics (PS-NPs) on the murine retina. This study revealed that PS-NPs can penetrate the blood–retina barrier, accumulate in retinal tissue, increase oxidative stress, and worsen light-induced photoreceptor degeneration [117]. However, as yet, evidence regarding the retinal effects of NPs is derived primarily from experimental studies, while epidemiological evidence in human populations remains limited.

Animal studies have shown that PS-NPs can penetrate the BRB and accumulate within retinal tissues, leading to increased oxidative stress and reduced dark-adapted electroretinographic responses, even in the absence of significant structural alterations. PS-NPs exposure also induces cytotoxicity and elevated ROS levels in photoreceptor cells, as well as oxidative stress in RPE cells. These effects are accompanied by gene and protein expression changes indicative of impaired phagocytic function and compromised cell junction integrity. Moreover, chronic PS-NPs exposure exacerbates light-induced photoreceptor degeneration and retinal inflammation—key pathophysiological features of AMD [117]. In vitro studies further demonstrated that PS-NP exposure significantly increases intracellular ROS levels in RPE cells. Excessive ROS accumulation depletes antioxidant defenses and induces oxidative damage to lipids and DNA, thereby impairing cellular integrity and function [118]. Moreover, PS-NPs trigger mitochondrial fragmentation and loss of membrane potential, characterized by upregulation of fission-associated proteins such as FIS1 and Drp1, along with increased expression of the autophagy marker LC3B, indicating enhanced mitophagic activity [118].

In summary, NPs exposure is strongly linked to retinal degenerative diseases such as AMD, through various molecular pathways, including oxidative stress, inflammation, DNA damage and repair dysregulation, and apoptosis. These findings underscore the importance of reducing environmental pollution and suggest potential targets for future therapeutic interventions. However, these findings are currently limited to experimental models, and it remains unclear whether the exposure levels and retinal accumulation observed under laboratory conditions accurately reflect human environmental exposure or AMD-specific effects.

Figure 5 summarizes the detrimental effects of pollutants on the retina that promote AMD progression.

## 4. Environmental Pollution and Retinal Vasculopathies

### 4.1. Diabetic Retinopathy (DR)

#### 4.1.1. General Aspects of DR

DR is a major ocular complication of diabetes and one of the leading causes of blindness worldwide, with a prevalence of approximately 30% to 40% among individuals with diabetes. More than 100 million people worldwide are currently affected by DR, which remains a leading cause of vision loss, particularly among working-age adults [119,120,121]. Epidemiological projections indicate that the global prevalence of DR will continue to rise sharply, increasing from 103 million people in 2020 to 130 million in 2030, and reaching an estimated 161 million by 2045 [122]. The dramatic increase in DR by more than 25% in just 10 years will likely strain healthcare systems. Consequently, DR imposes a substantial economic burden. In 2004, the direct healthcare costs associated with DR in the United States were estimated at $493 million annually [123]. Environmental factors appear to play a role in disease risk, with several studies demonstrating a clear association between higher levels of PM exposure and an increased incidence of DR [124,125].

DR is a microvascular complication of diabetes mellitus, primarily driven by chronic hyperglycemia and the accumulation of advanced glycation endproducts (AGEs). These pathological processes induce damage to the retinal microvasculature, resulting in impaired retinal perfusion and subsequent ischemia. In response, the retina attempts to compensate by forming new, fragile blood vessels, which are prone to leakage and may result in severe neovascular complications, including neovascular glaucoma [126]. Clinically, diabetic retinopathy can manifest with symptoms such as blurred vision, distorted vision, floaters, and, in more advanced cases, partial or complete vision loss [127]. Among eyes with moderate to severe non-proliferative diabetic retinopathy that do not receive preventive treatment, approximately 56.9% progress to vision-threatening proliferative retinopathy or central macular edema with associated vision loss within four years, underscoring the rapid deterioration in prognosis without timely intervention [128]. In Figure 6, a clinical example of diabetic retinopathy is presented.

#### 4.1.2. Epidemiological Associations Between Pollutant Exposure and DR

Growing evidence indicates that environmental pollution may contribute to the development and progression of DR, an increasingly relevant concern given the rising global prevalence of diabetes. In a retrospective population-based, nested case–control study conducted in Taiwan, higher PM exposure was positively associated with the risk of DR, with 26% and 28% higher odds observed among individuals exposed to higher PM levels compared with those exposed to lower levels. In contrast, no significant associations were observed between DR and traffic-related air pollutants, including CO and NO_2_. However, these findings should be interpreted cautiously in light of several study limitations. DR was identified solely on the basis of diagnostic and treatment codes, and information regarding disease severity was unavailable. Moreover, because participants were identified after the diagnosis of DR, the study design could not determine whether air pollution exposure contributed to disease onset. Therefore, the observed associations may reflect a relationship between air pollution exposure and DR progression rather than the initial development of the disease [129].

Similarly, a cross-sectional study of diabetic patients in rural China identified a significant relationship between ambient fine particulate matter (PM_2.5_) and DR. For each 10 μg/m^3^ increase in PM_2.5_, the adjusted odds ratio for DR rose by 1.41, indicating that particulate pollution may exacerbate retinal injury in diabetes via oxidative and inflammatory pathways. The stronger association observed among individuals who consumed alcohol may indicate effect modification by lifestyle or other individual-level factors. However, this finding also highlights the potential influence of environmental and individual confounders, which should be considered when interpreting these results [130].

A prospective study based on data from the UK Biobank further investigated the association between long-term exposure to air pollution and the development of microvascular complications among more than 17,000 participants with type 2 DM who were free of microvascular or macrovascular complications at baseline. In single-pollutant models, exposure to PM_2.5_, PM_10_, NO_2_, and NO_x_ was not significantly associated with an increased risk of DR. In contrast, higher exposure to these pollutants was associated with an increased risk of diabetic nephropathy and neuropathy. However, none of these associations remained statistically significant in multipollutant models, suggesting that the observed relationships may be influenced by co-exposure to multiple air pollutants and potential correlations between individual pollutants [131].

#### 4.1.3. Pathophysiological Impact of Pollutant Exposure in DR

Although the mechanisms linking air pollution to DR are not yet fully defined, current evidence supports several biologically plausible pathways. PM, particularly PM_2.5_, is known to induce oxidative stress and systemic inflammation, accompanied by elevated circulating cytokines such as TNF-α, vascular endothelial growth factor (VEGF), intercellular adhesion molecule-1 (ICAM-1), and IL-6 [132,133]. Elevated concentrations of transition metals including nickel, copper, and arsenic within PM fractions have also been associated with higher levels of inflammatory mediators such as IL-6 and VEGF, suggesting that the chemical composition of PM contributes substantially to its toxic potential [134]. These pathways are relevant to DR pathogenesis because VEGF plays a central role in microvascular dysfunction, retinal ischemia, and progression to proliferative disease [135]. In addition, exposure to particulate pollutants has been linked to endothelial dysfunction and atherosclerotic changes—pathological processes that overlap with the microvascular characteristics of DR. Evidence from both animal models and human studies indicates that pollutant-induced vascular injury may accelerate retinal microangiopathy in individuals with diabetes [134,136,137].

Overall, available research suggests that air pollutants, particularly PM_2.5_, NO_2_, and SO_2_, may exacerbate DR development by amplifying oxidative stress, promoting inflammatory signaling, and impairing microvascular integrity. Hence, reducing exposure to ambient pollution, especially in highly polluted environments, may therefore represent a meaningful strategy to mitigate DR risk. Continued investigation into pollutant-specific molecular pathways is essential for improving prevention and therapeutic approaches.

Figure 7 provides a schematic overview of the molecular effects of pollutant exposure on DR.

### 4.2. Retinal Vein Occlusion (RVO)

#### 4.2.1. General Aspects of RVO

RVO is one of the most common retinal vascular disorders and typically presents as painless vision loss [138]. The condition substantially affects patients’ quality of life, limiting daily functioning and imposing long-term visual burden [139]. Venous stasis is generally attributed to the compression of retinal veins by adjacent atherosclerotic arteries, highlighting the contribution of arteriosclerotic change to RVO pathogenesis [140]. Recent cohort data from a European population reported a 5-year cumulative incidence of 0.35% for branch retinal vein occlusion and 0.043% for central retinal vein occlusion, with affected individuals experiencing an approximately 2- to 4-fold increase in all-cause mortality [141]. The management of RVO relies heavily on anti-VEGF therapy, which represents a substantial economic burden due to high treatment costs and associated productivity losses [142]. Clinically, patients commonly present with sudden, painless monocular central vision blurring, metamorphopsia, and floaters [143]. Long-term, standardized anti-VEGF therapy can maintain significant visual improvement in most patients. However, inadequate follow-up or suboptimal treatment intensity is associated with deterioration of visual outcomes over time [144]. Figure 8 presents a clinical picture of branch retinal vein occlusion.

#### 4.2.2. Epidemiological Associations Between Pollutant Exposure and RVO

Recent evidence has linked exposure to certain air pollutants with an increased risk of RVO [79]. A retrospective cohort study conducted in Taiwan involving 855,297 participants examined the effects of long-term exposure to airborne hydrocarbons, including volatile organic compounds, on RVO incidence. Prolonged exposure to THCs and NMHCs was associated with a significantly increased risk of RVO. In single-pollutant models, hazard ratios rose with increasing concentrations of both THCs and NMHCs. Notably, these associations remained robust in multipollutant models, indicating that chronic exposure to airborne hydrocarbons independently elevates RVO risk. However, several limitations should be considered when interpreting these findings. The analysis did not adequately account for potentially important confounding factors, particularly serum parameters such as total cholesterol, which are established risk factors for RVO. In addition, exposure assessment was based on residential location and therefore may not accurately reflect individual-level exposure [79]. Given that only one epidemiological study has examined the association between environmental pollutant exposure and RVO to date, these findings should be interpreted cautiously and require confirmation in independent studies.

#### 4.2.3. Pathophysiological Impact of Pollutants in RVO

The mechanisms through which pollutants might contribute to RVO are not yet fully understood, but current evidence points to oxidative stress and inflammation as central pathways. Environmental exposures such as ambient air pollution, tobacco smoke, and traffic-related noise have been shown to elevate intracellular ROS levels, thereby inducing oxidative stress [30,145,146]. Increased ROS contributes to vascular endothelial dysfunction and promotes retinal inflammatory responses, both of which are relevant to venous stasis and thrombotic susceptibility in the retinal circulation [147]. Inflammatory cytokines, including IL-1β, TNF-α, and IL-6, also play a prothrombotic and proatherogenic role. Systemic inflammation mediated by these cytokines can enhance hypercoagulability by upregulating tissue factor expression and activating the extrinsic coagulation pathway. Concurrently, these mediators suppress tissue-type plasminogen activator activity, impairing fibrinolysis and facilitating thrombus formation at the systemic and microvascular levels [148].

Collectively, these pollutant-associated oxidative and inflammatory processes create a pathological environment conducive to endothelial injury, coagulation imbalance, and ultimately the development and progression of RVO. But there are no studies directly analyzing the impact of pollutants in RVO and providing direct evidence of underlying pathophysiological mechanisms.

Figure 9 provides a schematic overview of the pollutant-induced molecular mechanisms implicated in RVO.

### 4.3. Central Retinal Artery Occlusion (CRAO)

#### 4.3.1. General Aspects of CRAO

CRAO is a major cause of acute and often irreversible vision loss, with an estimated incidence of approximately 1 per 100,000 individuals [149,150]. Despite its rarity, CRAO imposes a substantial economic burden. An analysis of the U.S. Nationwide Inpatient Sample reported a median inflation-adjusted cost of USD 34,668 per hospitalization and national charges nearing USD 115 million in 2014, underscoring the significant healthcare impact of this ophthalmic emergency [151]. CRAO results from thrombotic or embolic obstruction of the central retinal artery, leading to ischemia of the retina and optic nerve head and causing severe visual impairment [152,153]. In a large-clinic-based cohort study, more than 70% of non-arteritic CRAO cases were attributed to emboli originating from atherosclerotic carotid plaques, highlighting the central role of systemic vascular disease in its pathogenesis [154]. Clinically, patients typically present with sudden, painless monocular vision loss, representing an ocular analog of cerebral stroke [155]. Prognosis remains poor. A 2024 Northern California cohort reported that 86% of 794 eyes maintained visual acuity of ≤20/200 three months after onset, demonstrating the persistently limited recovery achievable with current conservative management strategies [156]. Figure 10 presents a clinical example of CRAO.

#### 4.3.2. Epidemiological Associations Between Pollutant Exposure and CRAO

Growing evidence indicates that ambient air pollution may act as a potential trigger for CRAO. A large retrospective, population-based cohort study identifying patients with newly diagnosed CRAO within a representative database of 1000,000 patients from Taiwan reported a significant effect of short-term NO_2_ exposure on CRAO onset. The risk was highest after day 4–5 of elevated NO_2_ exposure in diabetic individuals. Among hypertensive individuals aged over 65 years, SO_2_ exposure produced an even more increased risk for CRAO observed just one day after SO_2_ levels increased. Importantly, the analysis accounted for exposure to multiple pollutants [157]. Similar associations were observed in a daily time-series analysis of 2272 newly diagnosed CRAO cases in Poland. The onset of CRAO was positively associated with short-term changes in daily concentrations of PM_10_, NO_2_, SO_2_, O_3_, and CO, as well as with air temperature, during the days preceding diagnosis. However, individual-level information, including age, sex, and comorbidities, was unavailable, limiting the interpretation of these findings [158]. In addition, a 15-year observational study of 432 patients identified seasonal variation in CRAO incidence, with a peak during winter and a decline during summer. The study further reported significant associations between CRAO incidence and higher concentrations of NO_2_ and PM_2.5_, with stronger associations observed at lower temperatures. However, relevant individual-level factors, including comorbidities, were not incorporated into the analysis. Therefore, these findings should be interpreted cautiously and require confirmation in future prospective studies with more comprehensive assessment of individual risk factors and pollutant exposure [159].

#### 4.3.3. Pathophysiological Impact of Pollutants in CRAO

Multiple mechanisms have been proposed to explain how air pollutants contribute to embolic events such as CRAO. Fine particulate matter (PM_2.5_) and gaseous pollutants including CO and NO_2_ can induce excessive generation of ROS systemically and within retinal tissues [30]. Elevated ROS levels directly damage vascular endothelial cells and reduce NO bioavailability, thereby impairing vasodilation and promoting vascular spasm [160]. An experimental model of retinal ischemia demonstrated marked increases in oxidative stress markers such as 3-nitrotyrosine and malondialdehyde, indicating that retinal ischemic injury might involve ROS accumulation [161]. Oxidative stress also triggers apoptosis of retinal neurons, disrupts the blood–retinal barrier, and exacerbates vascular permeability, contributing to retinal edema and no-reflow phenomena after ischemic insult [162]. Current epidemiological data linking environmental pollutants to CRAO remain limited and are constrained by important methodological limitations, such as inadequate adjustment for cardiovascular morbidities and other potential confounders. Additionally, direct mechanistic studies investigating the effects of pollutants on ischemic retinal diseases, such as CRAO, are lacking. Nevertheless, it is known that oxidative stress is a well-established contributor in the pathogenesis of ischemic injury, and several experimental studies have demonstrated that environmental pollutants enhance oxidative stress, providing a biologically plausible link between pollutant exposure and CRAO. Future studies, particularly controlled animal models simulating pollutant exposure and well-designed clinical investigations, are needed to clarify causal pathways and support the development of preventive strategies targeting pollution-related thrombotic risk.

## 5. Environmental Pollution and Retinal Detachment (RD)

### 5.1. General Aspects of RD

RD arises when the neurosensory retina separates from the RPE, disrupting phototransduction processes [163]. A systematic review published in 2024 including 274,836 cases across 33 countries estimated a global annual incidence of rhegmatogenous RD at 12.17 per 100,000, with an approximate increase of 5.4 per 100,000 per decade since 1997 [164]. National claims data from Canada (2023) showed mean first-year eye-care expenditures of CAD 8924 following pneumatic retinopexy and CAD 11,937 following pars plana vitrectomy, indicating a substantial per-patient economic burden [165]. According to a 2024 clinical update, patients typically present with acute onset of floaters, photopsia, and peripheral visual field loss that progresses centrally as separation advances [166]. Long-term data from the Scottish Retinal Detachment Study reported a median best-corrected visual acuity of 0.1 logMAR in successfully reattached eyes; 93% of macula-on and 65% of macula-off detachments retained driving-standard acuity, whereas re-detachment (14%) was associated with markedly poorer outcomes, indicating a generally favorable presentation—and procedure-dependent—prognosis [167]. Figure 11 presents a clinical picture of RD.

### 5.2. Epidemiological Association Between Environmental Pollution and RD

The association between environmental exposures and retinal detachment has increasingly been investigated, although the available evidence remains heterogeneous. Thermal exposure, in particular, may be associated with RD risk. A population-based study conducted in Quebec, Canada analyzed 14,302 patients who underwent inpatient procedures for RD between April and September from 2006 to 2013 [168].

The study suggests that higher ambient temperatures were associated with an increased risk of traction retinal detachment, but not with other types of retinal detachment. Associations were strongest at <75 years of age. Regarding the temperature level, the association was substantial at 25 °C compared with 15 °C: the odds of traction retinal detachment were approximately 2-fold to 2.7-fold higher, depending on age. Specifically, the OR was 2.71 for people younger than 55, 2.73 for those aged 55–64, and 1.98 for those aged 64–75. Importantly, these figures refer to the mean outdoor temperature during the preceding week, not necessarily to a single day’s maximum temperature. So, the study does not establish a specific temperature threshold at which retinal detachment occurs; rather, it indicates that moving from a weekly mean of 15 °C to 25 °C was associated with substantially higher odds of traction retinal detachment. The study design reduced potential confounding by assessing exposure at the individual level and accounting for other relevant meteorological parameters, including humidity, sunlight, and atmospheric pressure. However, the inability to account for underlying retinal diseases and the use of procedure dates rather than the actual onset of RD limit the interpretation of these findings.

Studies assessing seasonal variation across different geographic regions have similarly reported a higher incidence of RD during summer than during winter [169,170,171,172,173]. In a retrospective chart review of 211 patients with RD over a 13-year period, RD incidence was significantly higher during warmer seasons, and patients diagnosed during these periods were significantly younger than those diagnosed during colder seasons. However, no significant direct association was identified between RD incidence and individual weather variables, including ambient temperature. These findings should therefore be interpreted cautiously and highlight the need for larger studies incorporating more comprehensive exposure assessments [169]. A multicenter study from Spain involving 256 individuals with RD further suggested an association between solar radiation and rhegmatogenous RD, indicating that additional environmental factors may contribute to RD risk [171]. However, this potential association requires confirmation in independent studies. The influence of seasonal and meteorological factors on RD incidence was also evaluated in a systematic review of eight studies. Although most studies reported a higher incidence of RD during the summer months, some found no significant association between RD incidence and seasonal or weather-related variables. Moreover, no single meteorological factor consistently emerged as a significant predictor of RD incidence across the analyzed studies [174].

Collectively, these findings suggest a temperature-related susceptibility and underscore the need to evaluate the impacts of heatwaves and broader climatic shifts on retinal health.

### 5.3. Pathophysiological Impact of Environmental Pollution in RD

To date, the evidence of a potential effect of environmental factors is limited to epidemiological studies, but direct mechanistic studies analyzing pathophysiological mechanisms or structural retinal changes on the cellular level as a consequence of environmental factors are lacking. Nevertheless, there are some experimental studies that might provide mechanistic insights into retinal injury identifying environment-induced cellular and molecular alterations in retinal cells. For example, high-energy short-wave blue light (≈400–500 nm) has been a hot topic in ophthalmology research in recent years, since LED lighting and display technologies are increasingly prevalent in daily life. Blue light can directly reach the retina and cause photochemical damage, especially to the RPE and photoreceptors [175]. Experimental work demonstrates that brief blue light exposure markedly elevates intracellular ROS in ARPE-19 cells, promoting DNA double-strand breaks, mitochondrial dysfunction, and suppression of cell proliferation [176]. Prolonged exposure increases RPE apoptosis (annexin-V positivity at 36 h) and activates autophagy at 24–48 h, indicating a progression from oxidative DNA damage to initiation of apoptotic and mitochondrial-clearance pathways [176]. Blue light also triggers necroptosis, evidenced by HMGB1 release, RIPK3 accumulation, and MLKL phosphorylation, which amplifies local inflammation and weakens RPE–retina adhesion [175]. In parallel, aberrant activation of the Nrf2–SLC7A11–HMOX1 axis results in excess free-iron release and lipid peroxidation, driving ferroptosis and further destabilizing retinal structure [175]. However, these findings were obtained under experimental conditions, and it remains unclear whether these findings are transferable to humans or propose mechanisms involved in the pathogenesis of RD. Moreover, emissions from commonly used light sources generally remain below established ocular hazard limits, limiting the direct translational relevance of these experimental findings.

Experimental studies also revealed that particulate pollutants can exert outer-BRB-disruptive effects, since PM_2.5_ disrupts tight-junction proteins such as ZO-1 in the RPE, thereby compromising barrier integrity [60]. Several toxins, such as pesticides, including chlorpyrifos, have been shown to promote oxidative stress, cell damage, and consecutive apoptosis in retinal neurons [177,178]. Cadmium, another toxic environmental pollutant, exposure has been shown to induce relevant histopathological damage in retinal layers by promoting inflammation and induction of a dominant proapoptotic Bax/Bcl-2 balance [41]. Furthermore, co-exposure of cadmium and pesticides, such as imidacloprid, has been shown to potentiate the toxic effects of cadmium, aggravating apoptosis of retinal cells. Experimental models of RD revealed a potential role of oxidative stress and BRB properties for visual outcomes after RD, indicative of a potential role of these two factors in the pathogenesis of RD [179].

Taken together, these findings indicate that diverse environmental pollutants might induce oxidative-stress amplification, tight-junction disruption, programmed cell death, and inflammatory activation within the retina. But it remains to be proven whether these alterations may contribute to the development of RD.

## 6. Environmental Pollution and Retinoblastoma

### 6.1. General Aspects of Retinoblastoma

Retinoblastoma is a malignant retinal tumor that predominantly affects young children. Approximately two-thirds (63%) of cases are diagnosed before two years of age and 95% before five years, suggesting that perinatal factors may contribute substantially to disease onset [180]. The tumor arises from biallelic loss or mutation of the RB1 gene and occurs in two forms: heritable and sporadic. Heritable retinoblastoma accounts for 35–45% of cases and is typically bilateral; it results from a germline RB1 mutation, usually inherited from the father or arising de novo in parental germ cells. The pathogenesis of retinoblastoma can be divided into two types: heritable and sporadic. Heritable cases account for approximately 35–45%, and almost all of them present as bilateral disease. Sporadic cases comprise 55–65% of diagnoses and are predominantly unilateral [181]. In hereditary cases, a defective allele is inherited from one parent, most often the father [182], due to hereditary or de novo mutations in the germline cells. These cases frequently present as bilateral disease. A 2025 Global Burden of Disease-based assessment covering 204 countries estimated 6275 new retinoblastoma cases in 2021, corresponding to an age-standardized incidence of 0.094 per 100,000 children under 10 years and an average annual increase of 1.35% since 1990 [183]. Recent cost analyses indicate substantial economic burdens. Treatment in China typically incurs USD 27,815 in direct medical costs per child [184], whereas in a U.S. tertiary center, first-year expenses average USD 301,151 for globe-salvage therapy compared with USD 104,764 for primary enucleation [185], underscoring the substantial—and highly variable—global economic burden. Prognostic differences remain pronounced. A 2024 multicenter cohort study of 491 patients from 23 countries in the Americas reported three-year overall survival of 99% in high-income settings versus 60% in low-income settings, underscoring persistent global disparities despite advances in therapy [186].

### 6.2. Epidemiological Association Between Environmental Pollution and Retinoblastoma

Environmental exposures, particularly parental tobacco smoke, have been investigated as potential contributors to sporadic (non-hereditary) retinoblastoma. A large multicenter case–control study, involving 488 cases of retinoblastoma assessed whether parental smoking or alcohol consumption before and after conception was associated with unilateral or bilateral sporadic retinal tumors. In unmatched analyses, smoking more than 10 pack-years and smoking more than 10 cigarettes per day in the year before pregnancy were associated with an increased risk of bilateral retinoblastoma. But this observation was not approved in matched analyses. Furthermore, residual confounding by socioeconomic status could not be excluded in this study [187]. Broader evidence regarding childhood cancers comes from the Air Pollution and Childhood Cancer Study, a case–control study conducted in California involving more than 4000 cases. This study investigated whether prenatal exposure to air pollution was associated with childhood malignancies, including bilateral retinoblastoma. Using land-use regression models to estimate in utero exposure to traffic-related pollutants, the study reported that each 25 ppb increase in pollutant concentration during the third trimester was associated with a 15% and 13% increase in the odds of bilateral retinoblastoma for NO and NO_2_, respectively. No significant associations were observed for unilateral retinoblastoma. However, exposure estimates based solely on residential address at birth may have resulted in exposure misclassification due to residential mobility. In addition, postnatal environmental exposures could not be accounted for, further limiting the interpretation of these findings [188]. Perinatal exposure to specific volatile and combustion-related pollutants has also been investigated. Maternal exposure to benzene, toluene, 1,3-butadiene, ethylbenzene, and xylene—compounds largely emitted from gasoline and diesel combustion—was associated with increased risk of retinoblastoma [189]. Associations were also reported for exposure to chloroform, chromium, parachlorobenzene, nickel, and acetaldehyde during pregnancy or in the child’s first year of life [189]. However, in this large case–control study conducted among California children, which included 103 cases of retinoblastoma and more than 30,000 controls, exposure misclassification could not be excluded. Furthermore, residual confounding, particularly by maternal smoking, could not be ruled out [189].

Another area of investigation concerns early-life exposure to per- and polyfluoroalkyl substances (PFAS) and the risk of retinoblastoma. In a study including 501 children with retinoblastoma, higher neonatal concentrations of perfluorooctanesulfonic acid (PFOS) at birth were associated with 29% higher odds of retinoblastoma overall and 42% higher odds of unilateral disease [190]. The strongest associations were observed among children of Mexican-born mothers. Among children of U.S.-born mothers, higher PFOS concentrations were associated with a 15% increase in the odds of unilateral retinoblastoma per quartile increase and a 71% increase among those with above-average PFOS concentrations. Similarly, higher perfluorooctanoic acid (PFOA) exposure was associated with an increased risk of retinoblastoma in this subgroup. The authors proposed that differences between children of U.S.-born and Mexican-born mothers may partly reflect differences in dietary exposure patterns [190].

### 6.3. Pathophysiological Impact of Environmental Pollution in Retinoblastoma

Cadmium (Cd) is a pervasive environmental heavy metal with demonstrated cytotoxic and regulatory effects in retinoblastoma (RB) cells. In Y79 human RB cells, exposure to 15 μM CdCl_2_ significantly downregulated RB1 and the oncogene N-MYC, while inducing marked upregulation of HSP70 and metallothionein genes, reflecting a robust cellular stress response [191]. These findings suggest that cadmium promotes oxidative stress, activates protective transcriptional programs, and perturbs cell-cycle and proliferative signaling pathways [191]. However, these observations do not demonstrate a role of cadmium in retinoblastoma initiation. Volatile organic compounds (VOCs) and industrial solvents might also have an effect on retinoblastoma. Prenatal exposure to benzene, toluene, ethylbenzene, xylene, and 1,3-butadiene—key constituents of vehicle emissions and industrial effluents—has been associated with significantly increased RB risk in offspring [189]. Many of these agents are classified by the International Agency for Research on Cancer (IARC) as probable or known human carcinogens. Their carcinogenicity frequently involves metabolic activation to electrophilic intermediates capable of forming DNA adducts, inducing point mutations, and causing chromosomal breaks. Benzene metabolites can drive chromosomal translocations, whereas 1,3-butadiene metabolites induce gene mutations, providing plausible genotoxic mechanisms [192]. Direct mechanistic evidence linking environmental pollutants to retinoblastoma initiation is currently scarce. Most available experimental studies have investigated the response of established retinoblastoma cell lines rather than the early stages of tumorigenesis. Endocrine-disrupting chemicals such as bisphenol A (BPA) further demonstrate molecular disruption in RB cells. Chronic low-dose BPA exposure induces extensive transcriptomic and proteomic reprogramming [193]. RNA sequencing analyses show downregulation of genes related to cell-cycle control and upregulation of genes involved in post-transcriptional regulation [193]. In particular, BPA induced abnormal alternative splicing events, such as the detection of abnormal intron retention in the mRNAs of MAGOH homolog B (MAGOHB) and heterogeneous nuclear ribonucleoprotein D (HNRNPD) genes. Proteomics profiling identified nine differentially expressed proteins, including upregulated molecular chaperones and stress-related proteins, such as oxygen-regulated protein (HYOU1), protein disulfide isomerase (PDIA5), etc., and metabolic and nucleic acid-binding proteins, such as Methylenetetrahydrofolate dehydrogenase (NADP^+^ dependent) 2, mitochondrial (MTHFD2), TATA-box binding protein (TBP), etc. [193]. These alterations indicate that chronic BPA exposure perturbs transcriptional regulation, splicing fidelity, and metabolic homeostasis, potentially leading to impaired proliferative signaling, cell-cycle dysregulation, and mild endoplasmic reticulum stress [193].

In summary, despite their distinct sources, cadmium, VOCs, and endocrine disruptors such as BPA converge mechanistically through DNA damage and transcriptional/splicing reprogramming, downregulation of tumor-suppressive pathways (including RB1), and disruption of cell-cycle, proliferative, and metabolic networks. Collectively, these findings provide mechanistic insight into how environmental pollutants may influence the biology of established retinoblastoma cells. However, whether these molecular alterations contribute to retinoblastoma initiation or progression in vivo remains unknown.

## 7. Environmental Pollution and Retinal Developmental Disorders

### 7.1. General Aspects of Retinal Developmental Disorders

Retinal developmental disorders encompass structural or functional abnormalities of the retina arising during embryogenesis or the early postnatal period. Common forms include retinopathy of prematurity (ROP), retinal dysplasia, and congenital pigmentary retinopathy. ROP remains a leading cause of blindness in infants and young children, with a global prevalence of approximately 32% among preterm infants [194]. Severe ROP accounts for about 7.5% of cases and can result in profound visual loss [194]. Clinical manifestations vary with the extent of retinal injury and may include decreased visual acuity, visual field defects, night blindness, or complete blindness. Beyond lifelong visual impairment, these conditions impose considerable medical and socioeconomic burdens. Globally, ROP-related vision loss leaves tens of thousands of infants permanently disabled each year, resulting in substantial long-term costs for families and healthcare systems [195].

### 7.2. Epidemiological Association Between Environmental Pollution and Retinal Developmental Disorders

Maternal exposure to air pollution during pregnancy can affect fetal retinal development through placental transfer mechanisms. A cross-sectional observational NICU-based study in India involved 196 screened preterm neonates reported that exposure to household stove smoke during pregnancy—particularly from cooking with high-pollution fuels—significantly increased the incidence of ROP among preterm infants. The risk of ROP was further elevated in households with indoor smoking or the use of biomass fuels for cooking [196].

In addition, a large cross-sectional study involving 59,054 schoolchildren conducted in Guangdong, China, found a positive association between long-term exposure to fine particulate matter (PM_2.5_) and the risk of visual impairment in children, suggesting that particulate air pollution may adversely affect retinal function. However, these findings were only based on visual acuity testing and did not differentiate between the ocular reasons for visual impairment. Hence, future studies are required to specify reasons for visual impairment [197].

Prenatal exposure to heavy metals might also be toxic to neurovisual development. Experimental animal studies further revealed that prenatal lead exposure increases rod cell counts and reduces dopamine synthesis in the adult offspring retina, indicating disrupted neurotransmitter balance and retinal homeostasis even at low exposure levels [198]. Findings from the Canadian MIREC cohort with around 500 mother–infant pairs demonstrated that maternal blood levels of lead and mercury were negatively correlated with infants’ visual evoked potentials (VEPs), showing delayed responses. Notably, even low levels of mercury exposure were associated with subtle visual deficits. However, the effect of potential additive and or synergistic effects of unmeasured environmental pollutants could not be excluded [199].

Evidence linking disinfection byproducts such as chloroform to retinal developmental abnormalities in humans remains scarce; however, toxicological studies in rodents have shown that trihaloacetic acid (TCA) can induce ocular malformations, including microphthalmia and anophthalmia, in fetal rats [200]. Other persistent organic pollutants, including polychlorinated biphenyls (PCBs) and polybrominated diphenyl ethers (PBDEs), also lack sufficient epidemiological data regarding visual development.

Prenatal exposure to pesticides, particularly organophosphates, has been associated with impaired visual function in infants. A Chinese birth cohort measuring 30 organophosphate pesticides in cord blood reported that chlorpyrifos exposure was significantly associated with reduced grating visual acuity (VA) scores in 9-month-old infants, suggesting that prenatal chlorpyrifos exposure may disrupt the development of the visual pathway and could lead to mild early visual dysfunction. Other detected organophosphates, such as phorate, were negatively correlated with head circumference in infants, though their association with visual outcomes requires further investigation [201].

### 7.3. Pathophysiological Impact of Environmental Pollution in Retinal Developmental Disorders

Retinal organoids are capable of recapitulating the morphological formation and cellular differentiation processes of the human embryonic retina in vitro, and are therefore regarded as an important model system for studying the developmental toxicity of environmental pollutants [202]. Studies have shown that PM_2.5_ exposure in retinal organoid culture systems induces the accumulation of ROS, leading to an imbalance in the PI3K/Akt and MAPK signaling pathways and suppressing FGF8/FGF10-mediated early neuroretinal differentiation [92]. Recent studies have indicated that lead-based perovskite nanoparticles (Pb-PNPs), which are widely used in the photovoltaic industry, exert developmental toxicity on retinal organoids by upregulating Caspase-3 to promote apoptosis and downregulating Pax6 to inhibit the development of retinal ganglion cells [203]. Recent studies have demonstrated that polystyrene nanoplastics (PS-NPs) exhibit size-, dose-, and developmental stage-dependent toxicity in human neural retinal organoids (hNROs), with smaller particles (100 nm) and higher concentrations causing the most severe developmental impairments [204]. The findings further reveal that PS-NPs disrupt the MAPK, PI3K/Akt, Calcium, HIF-1, and Wnt signaling pathways, thereby markedly suppressing cell proliferation and promoting apoptosis, accompanied by reduced organoid size, impaired neuronal differentiation, and depletion of retinal ganglion cells [204]. Moreover, co-exposure with cadmium (Cd) significantly exacerbates PS-NP-induced retinal toxicity, suggesting that the synergistic interaction between nanoplastics and heavy metals in the environment may aggravate the risk of retinal developmental disorders in fetuses and infants [204]. A study on cadmium revealed that it disrupts retinal development through mechanisms involving metal ion competition, oxidative stress, mitochondrial dysfunction, and activation of the AP-1 (FOS/JUN) signaling pathway, providing molecular evidence for the risk of retinal neurodevelopmental impairment associated with prenatal environmental cadmium exposure [38].

In summary, diverse environmental pollutants converge on common mechanistic pathways—oxidative stress, mitochondrial dysfunction, and dysregulation of PI3K/Akt, MAPK, Wnt, and calcium signaling—resulting in impaired neuroretinal differentiation, reduced proliferation, and increased apoptosis. Co-exposure scenarios, particularly involving nanoplastics and cadmium, demonstrate synergistic toxicity, underscoring the heightened susceptibility of the developing retina to complex environmental mixtures during fetal and early postnatal life.

## 8. Environmental Pollution and Glaucoma

### 8.1. General Aspects of Glaucoma

Glaucoma is a prevalent neurodegenerative disease characterized by chronic and progressive degeneration of retinal ganglion cells and their axons. Clinical manifestations include pathological excavation of the optic nerve head and characteristic visual field defects. With an estimated global prevalence of 3.54%, glaucoma is a leading cause of irreversible blindness worldwide, and the number of affected individuals is expected to increase with the aging population [205]. Elevated intraocular pressure remains the only established modifiable risk factor and results from an imbalance between aqueous humor production and its drainage through the trabecular meshwork and uveoscleral pathways. However, glaucomatous damage can also occur in individuals with normal IOP, indicating that additional pressure-independent mechanisms may contribute to disease pathogenesis, including impaired ocular blood flow, vascular dysfunction, inflammation, and oxidative stress [206]. Nevertheless, lowering IOP through topical medications or surgical interventions remains the primary therapeutic strategy for slowing disease progression [207].

### 8.2. Epidemiological Association Between Environmental Pollution and Glaucoma

Several epidemiological studies have reported associations between exposure to ambient air pollutants, particularly PM_2.5_, PM_10_, NO_2_, and CO, and an increased risk of glaucoma and disease progression. In the UK biobank cohort, 481,113 participants were followed for approximately 12 years. Each interquartile-range increase in PM_2.5_ exposure was associated with a 3% higher risk of incident glaucoma, while individuals in the highest exposure quartile had a 10% higher risk compared with those in the lowest quartile. However, air pollution exposure was estimated solely on the basis of participants’ residential addresses; therefore, indoor exposure and exposure occurring at workplaces or other locations were not captured and may have resulted in exposure misclassification [208]. Notably, the association between long-term air pollution exposure and glaucoma risk was more pronounced among individuals with a high genetic predisposition, as assessed using polygenic risk scores [209]. Comparable findings were reported in a Nationwide Cohort Study from Taiwan including 590,338 individuals aged >20 years. During a 10-year observation period, higher average pollutant concentrations were associated with an increased risk of primary open-angle glaucoma, with the strongest association observed for CO [210]. Although several pollutant concentrations exceeded international air-quality standards in this study, associations with glaucoma were also observed at exposure levels below WHO guideline thresholds, including for SO_2_ and CO. These findings suggest that chronic exposure to pollutant concentrations below current guideline levels may still be associated with adverse ocular outcomes [210]. The China Health and Retirement Longitudinal Study (CHARLS) further analyzed the relationship between long-term exposure to six major air pollutants and glaucoma in 33,147 participants. Annual mean pollutant concentrations were estimated according to participants’ residential locations. Higher ambient concentrations of PM_10_ and PM_2.5_ were associated with a greater prevalence of glaucoma, and dose–response analyses indicated pollutant-specific, nonlinear associations between exposure and glaucoma risk [211]. However, glaucoma diagnosis was based solely on self-reported information without confirmation by objective ophthalmological examinations. This limitation may have resulted in underdiagnosis, particularly given the absence of routine population-based glaucoma screening in China [211]. Data from the same cohort further indicated that ambient temperature was not significantly associated with glaucoma incidence. In contrast, NO_2_ and PM_2.5_ were identified as significant environmental risk factors, with NO_2_ showing a short-term association with increased glaucoma risk during the first days following exposure [212]. Interestingly, the association was stronger among smokers, suggesting that individual lifestyle factors may modify susceptibility to the potential effects of air pollution [212]. In a clinical study involving 120 participants with primary open-angle glaucoma, standard ophthalmological parameters were assessed together with air pollution data, including PM_2.5_ concentrations and the air quality index, over a three-year period. Higher average PM_2.5_ exposure over the preceding one and three months was associated with higher intraocular pressure and lower visual field mean sensitivity. In contrast, PM_2.5_ concentrations measured on the day of examination were not significantly associated with these parameters, suggesting that cumulative rather than acute exposure may be more relevant to glaucomatous changes. Furthermore, three-month average PM_2.5_ exposure was significantly associated with disease severity and was identified as an independent risk factor for glaucoma [213]. These findings require confirmation in larger, multicenter studies incorporating standardized individual-level exposure assessment and detailed information on lifestyle and behavioral factors.

### 8.3. Pathophysiological Impact of Environmental Pollution in Glaucoma

Experimental studies provide mechanistic evidence supporting a causal relationship between environmental pollutants and glaucomatous changes. In mice exposed to elevated concentrations of PM_2.5_, intraocular pressure (IOP) increased as a result of increased resistance to aqueous humor outflow, representing a potential mechanism contributing to ocular hypertension. Increased levels of oxidative stress markers, including 3-nitrotyrosine, were detected in conventional aqueous humor outflow tissues, suggesting that protein nitration may contribute to impaired outflow and PM_2.5_-induced ocular hypertension [214]. Furthermore, exposure to particulate matter induced activation of the NLRP3 inflammasome in aqueous humor outflow tissues, accompanied by stimulation of downstream pro-inflammatory pathways involving interleukin-1β and caspase-1 [215]. Consistent with these findings, in vitro exposure of human trabecular meshwork cells to particulate matter reduced cell viability and impaired cellular contractility. Oxidative stress- and inflammation-associated pyroptosis of trabecular meshwork cells was identified as a potential mechanism underlying particulate matter-induced ocular hypertension [215]. In addition to elevated IOP, retinal neurodegeneration, particularly degeneration of retinal ganglion cells, is a central feature of glaucoma. Following six months of particulate matter exposure in mice using a versatile aerosol concentration enrichment system, electroretinography demonstrated reduced light responses. These functional changes were accompanied by retinal neurodegeneration, including retinal thinning and reduced expression of retinal ganglion cell-specific markers [216]. Increased pro-inflammatory cytokine production and oxidative stress-associated DNA damage were identified as potential underlying mechanisms [216]. The direct toxicity of particulate matter to retinal cell populations, particularly retinal ganglion cells, has also been demonstrated in retinal organoids. Particulate matter exposure suppressed cell proliferation and promoted apoptosis through activation of the MAPK and PI3K/Akt signaling pathways, resulting in cellular disorganization and structural abnormalities, including altered retinal ganglion cell distribution [92]. In addition to particulate matter, cigarette smoke extract induced RGC injury in an in vitro study of primary rat retinal ganglion cells, with apoptosis and autophagy identified as contributing mechanisms [217].

## 9. Conclusions

Environmental exposures from diverse sources contribute to a spectrum of retinal diseases, highlighting the complexity and heterogeneity of their underlying pathophysiology. This review synthesizes current evidence on how distinct classes of environmental pollutants affect retinal structure and function and outlines the molecular pathways through which these exposures exert their effects. To date, PM_2.5_ and NO_2_ have the most consistent epidemiological evidence of adverse effects on retinal health, particularly in relation to AMD. However, also numerous other environmental factors interact with multiple cellular and signaling networks to influence retinal vulnerability and disease progression. These insights have important implications for public health, offering direction for monitoring key environmental variables, identifying at-risk populations, and addressing disparities in retinal health outcomes. However, important limitations of the current evidence need to be considered. Most epidemiological studies are observational and therefore cannot establish causality. Exposure estimates are frequently derived from residential addresses or area-level models and may not adequately capture individual exposure, indoor environments, occupational exposure, mobility, or cumulative lifetime exposure. Furthermore, residual confounding by age, smoking, socioeconomic status, comorbidities, lifestyle, genetic susceptibility, and other environmental exposures cannot always be excluded. Heterogeneity in disease definitions, exposure assessment, outcome ascertainment, and statistical modeling further complicates comparisons between studies. Several reported associations were also attenuated or disappeared in multipollutant models, emphasizing the importance of considering correlated exposures and potential co-exposure effects.

Future research should therefore prioritize prospective, multicenter studies incorporating standardized individual-level exposure assessment, longitudinal follow-up, and objective ophthalmological endpoints. Integration of personal exposure monitoring with geospatial and biomarker-based approaches may improve characterization of cumulative and biologically relevant exposure. Special attention should also be given to susceptible populations, including individuals with diabetes, vascular disease, genetic predisposition, or pre-existing retinal pathology. Mechanistic studies should further clarify the contribution of oxidative stress, inflammation, vascular dysfunction, and other molecular pathways and determine whether these processes represent common mechanisms across different ocular diseases.

Overall, current evidence supports a potential role of environmental exposures in ocular and retinal disease, while highlighting substantial gaps in the understanding of exposure–response relationships and causality. A more integrated approach combining epidemiological, experimental, and mechanistic evidence will be essential to determine the extent to which environmental pollution contributes to ocular disease burden and to identify potentially modifiable environmental risk factors for prevention and disease management.

## Figures and Tables

**Figure 1:**
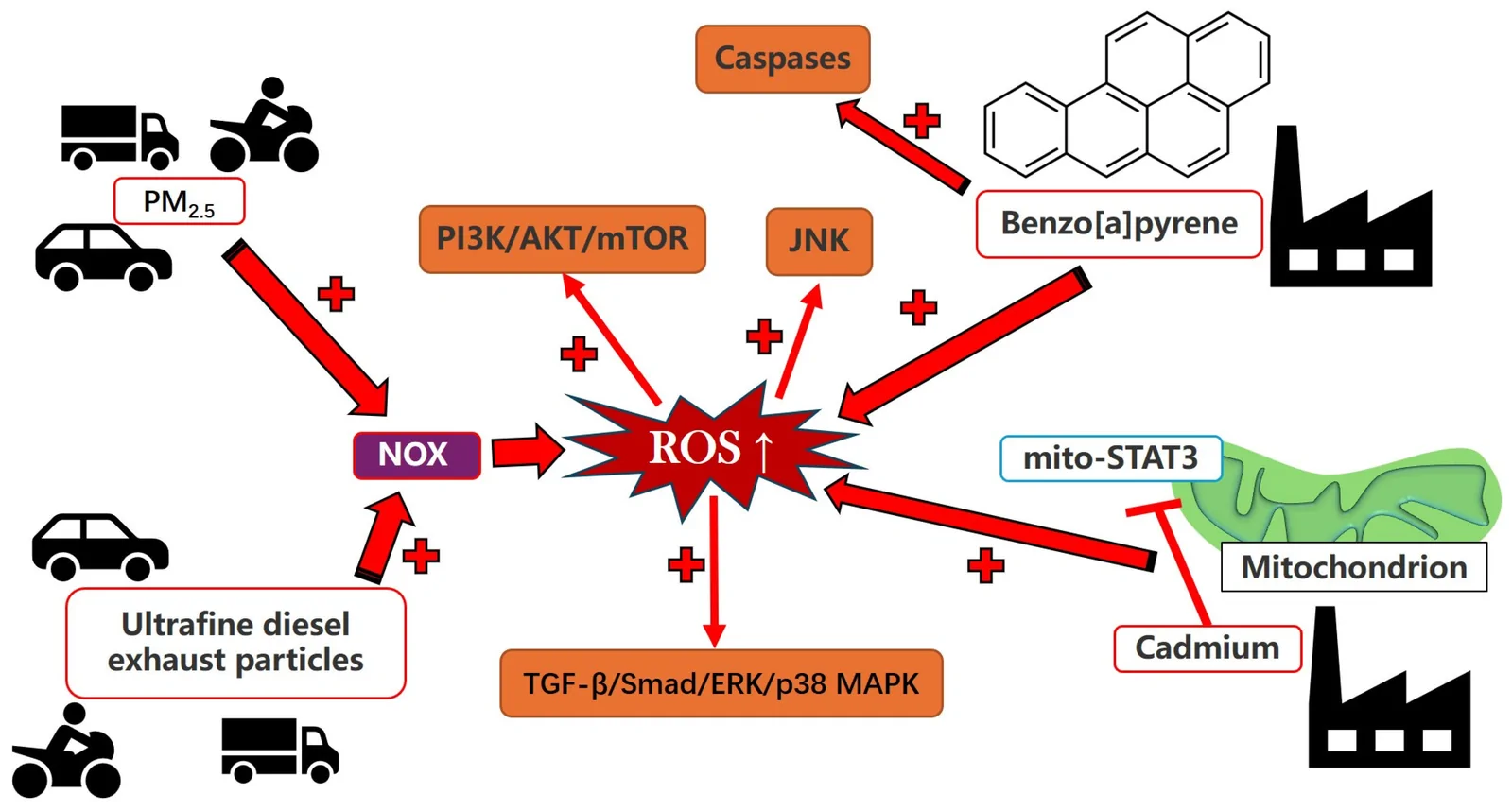
Illustration of the pathogenic mechanisms induced by pollutants that lead to oxidative stress. PM_2.5_ activates NOX eventually leading to an increase in reactive oxygen species (ROS). Diesel exhaust particles also promote excessive expression of ROS by activating NOX. Benzo[a]pyrene promotes ROS generation and activates caspases. Likewise, cadmium promotes generation of ROS. These pathways converge together, significantly increasing intracellular ROS levels, which further activate downstream signaling pathways.

**Figure 2:**
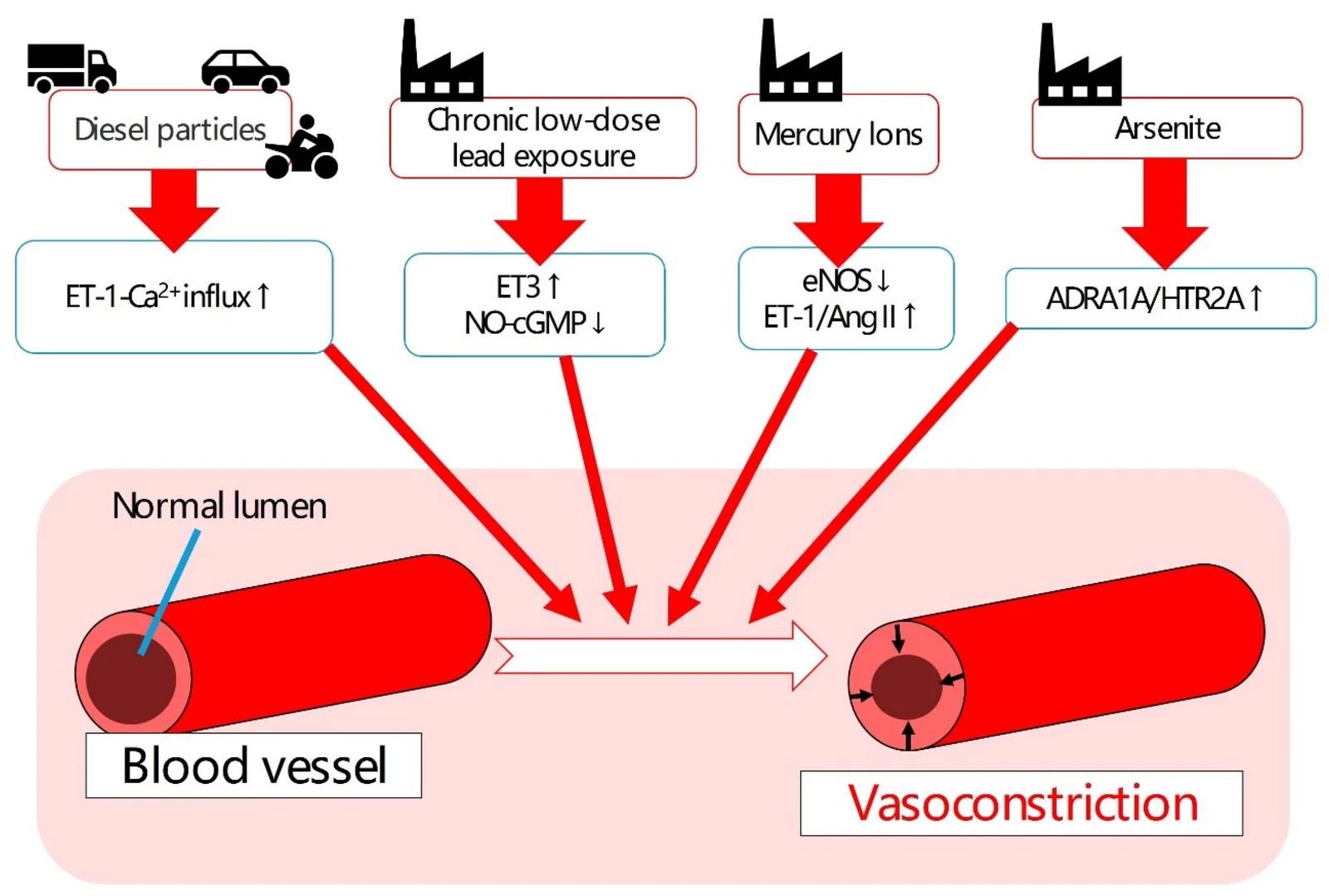
Scheme of the molecular pathways activated by pollutants and leading to vasoconstriction. Diesel exhaust particles promote the release of endothelin-1 (ET-1) from endothelial cells. This process is mediated by the ETA receptor, leading to calcium ion (Ca^2+^) influx and the activation of myosin light chain kinase (MLCK), which subsequently causes acute vasoconstriction. Low-dose lead upregulates endothelin-3 (EDN3) and inhibits the NO-cGMP relaxation axis, allowing the contraction signal to remain dominant. On the one hand, mercury ions inhibit eNOS to reduce NO production; on the other hand, they increase the ET-1/Ang II level, thereby doubly enhancing the contractile pathway. Arsenite increases the phosphorylation of smooth muscle myosin light chain (p-MLC) through the ADRA1A and HTR2A pathways, amplifying the contractions induced by neurotransmitters and hormones.

**Figure 3:**
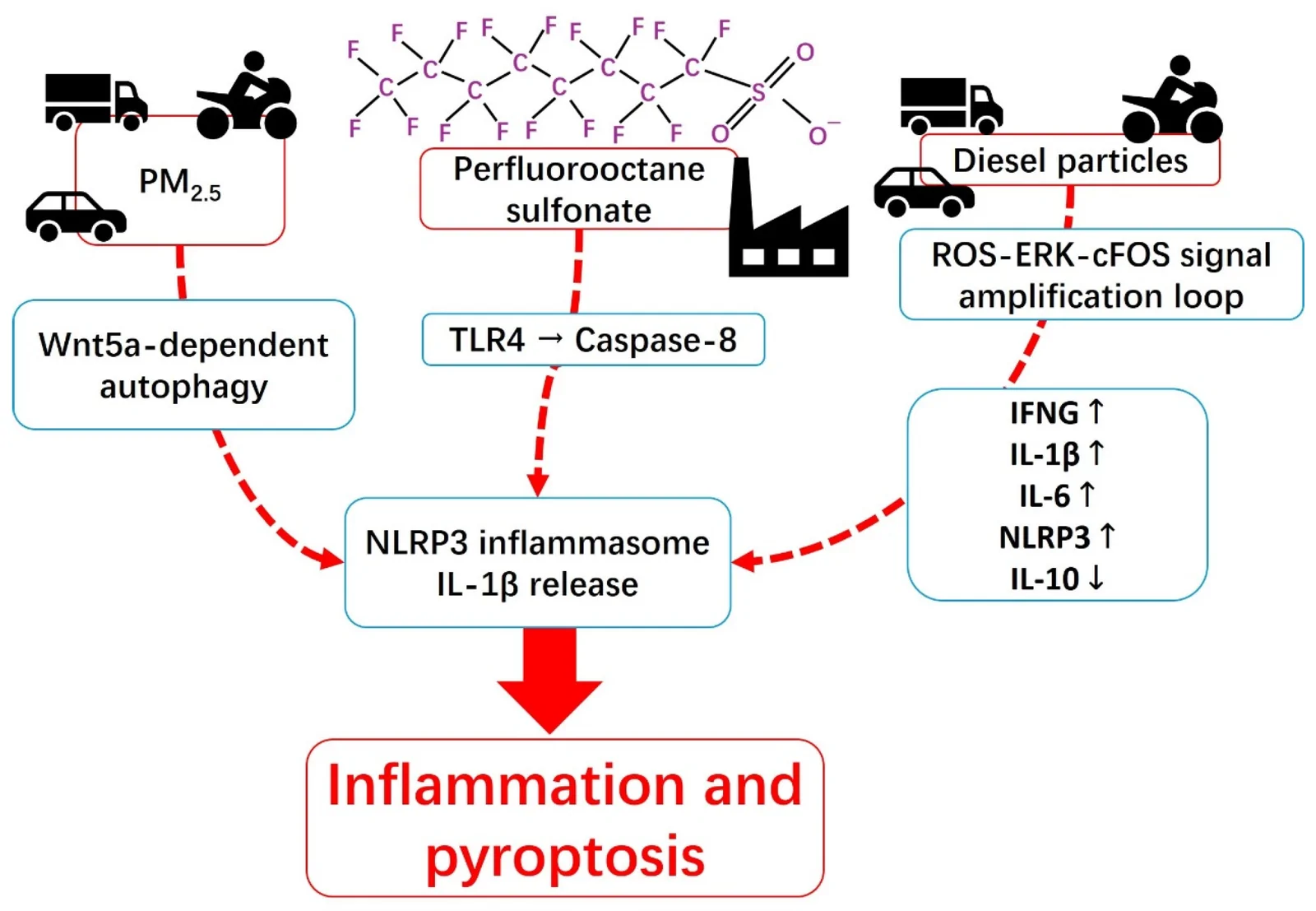
Signaling mechanisms by which environmental pollutants induce inflammation. PM_2.5_ activates Wnt5a-dependent autophagy, subsequently initiating NLRP3 inflammasome activation and promoting IL-1β release. Diesel exhaust particulates amplify inflammation through the ROS–ERK–cFOS signaling loop, upregulating IFN-γ, IL-1β, IL-6, and NLRP3 while suppressing the anti-inflammatory cytokine IL-10. PFOS directly engages the TLR4–caspase-8 pathway, leading to caspase-1-mediated maturation of IL-1β.

**Figure 4:**
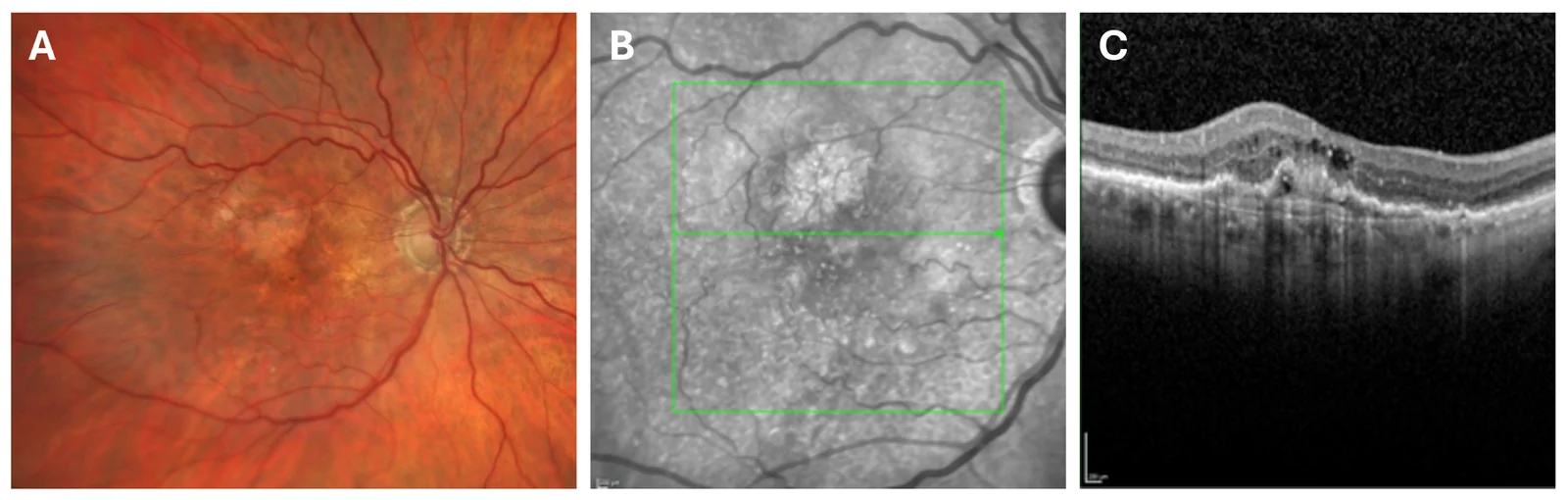
Fundus photograph of the right eye of a 67-year-old patient with macular drusen and scarring in neovascular age-related macular degeneration (**A**). Optical coherence tomography (OCT) shows related intraretinal fluid and detachment of the retinal pigment epithelium in the cross-section (**B**,**C**). The green rectangular box (**B**) indicates the area selected for OCT analysis. The green line within the box represents the segmentation line reflecting the scan position of the scan presented in (**C**).

**Figure 5:**
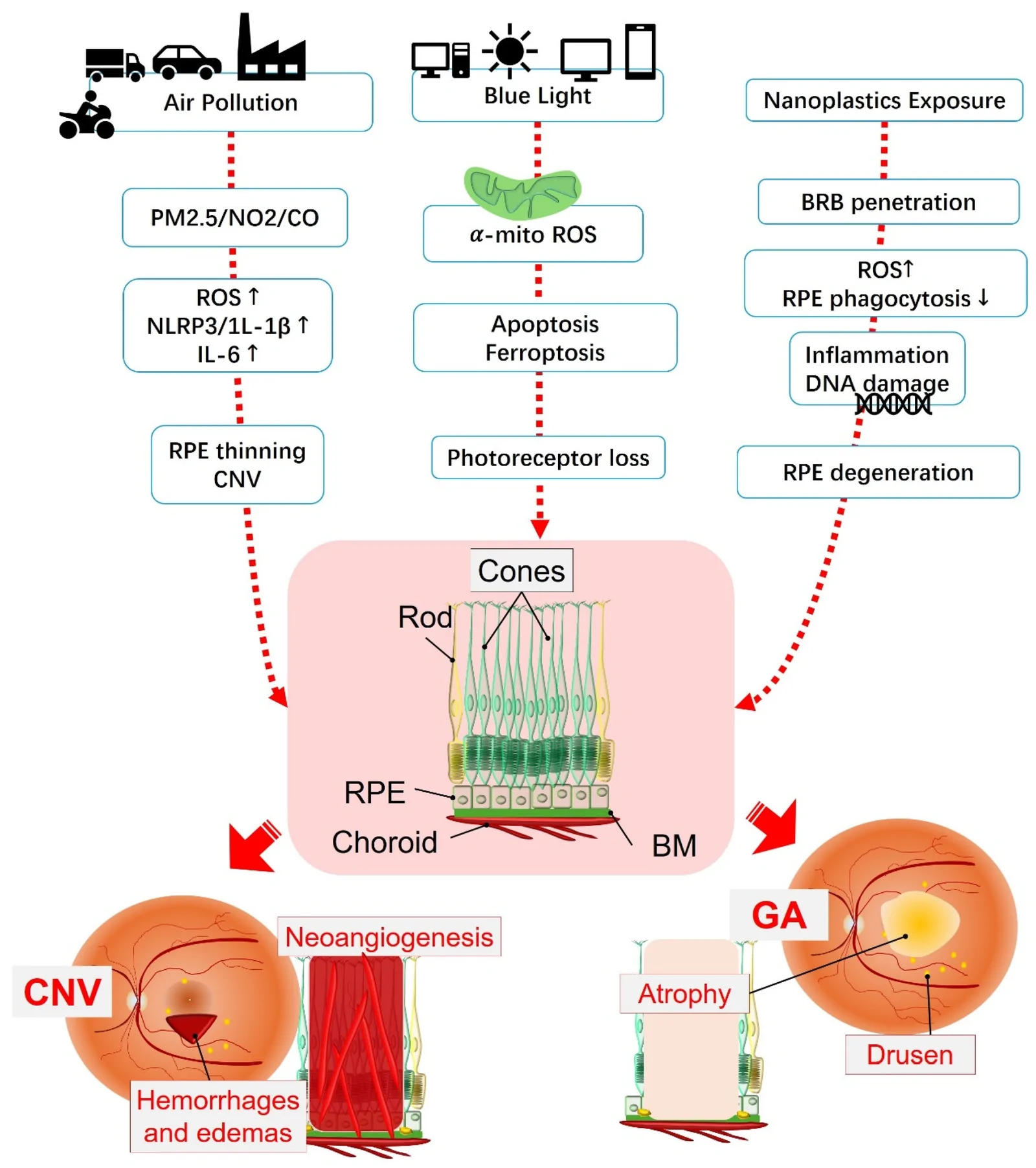
Air pollution (PM_2.5_/NO_2_/CO) generates excessive ROS through NADPH oxidase (NOX), activates NLRP3 inflammasome and pro-inflammatory factors (IL-1β, IL-6), and simultaneously disrupts miRNA expression, leading to thinning of the RPE layer and choroidal neovascularization (CNV). Light pollution induces a surge in mitochondrial ROS within the retina, and activates p53/Caspase-3-mediated apoptosis and GSH/GPX4-regulated ferroptosis, ultimately leading to the loss of photoreceptor cells. Nanoplastics enter the retina through the blood–retinal barrier (BRB), increasing reactive oxygen species (ROS), weakening the phagocytic function of retinal pigment epithelial cells (RPEs), and triggering inflammation and DNA damage, leading to degeneration of the RPE and photoreceptors.

**Figure 6:**
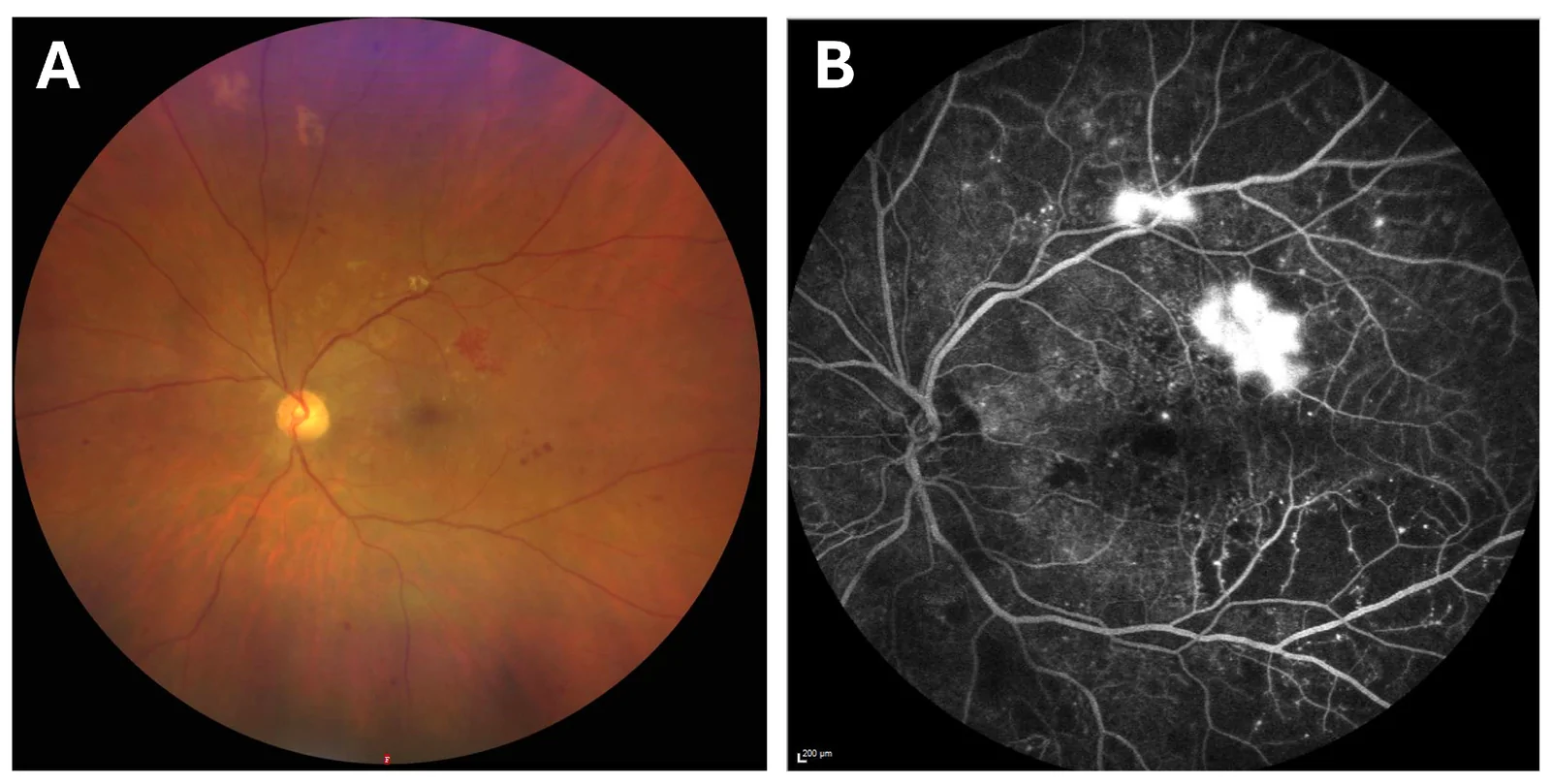
Proliferative diabetic retinopathy of the left eye in a 41-year-old patient with type 1 diabetes. In the fundus photograph hemorrhages in all four retinal quadrants and macular exsudates are visible (**A**). Fluorescein angiography shows retinal microaneuymsms (white dots), capillary non-perfusion (dark areas) and pathologic neovascularization with vascular leakage (bright areas) as a consequence of progressive retinal ischemia (**B**).

**Figure 7:**
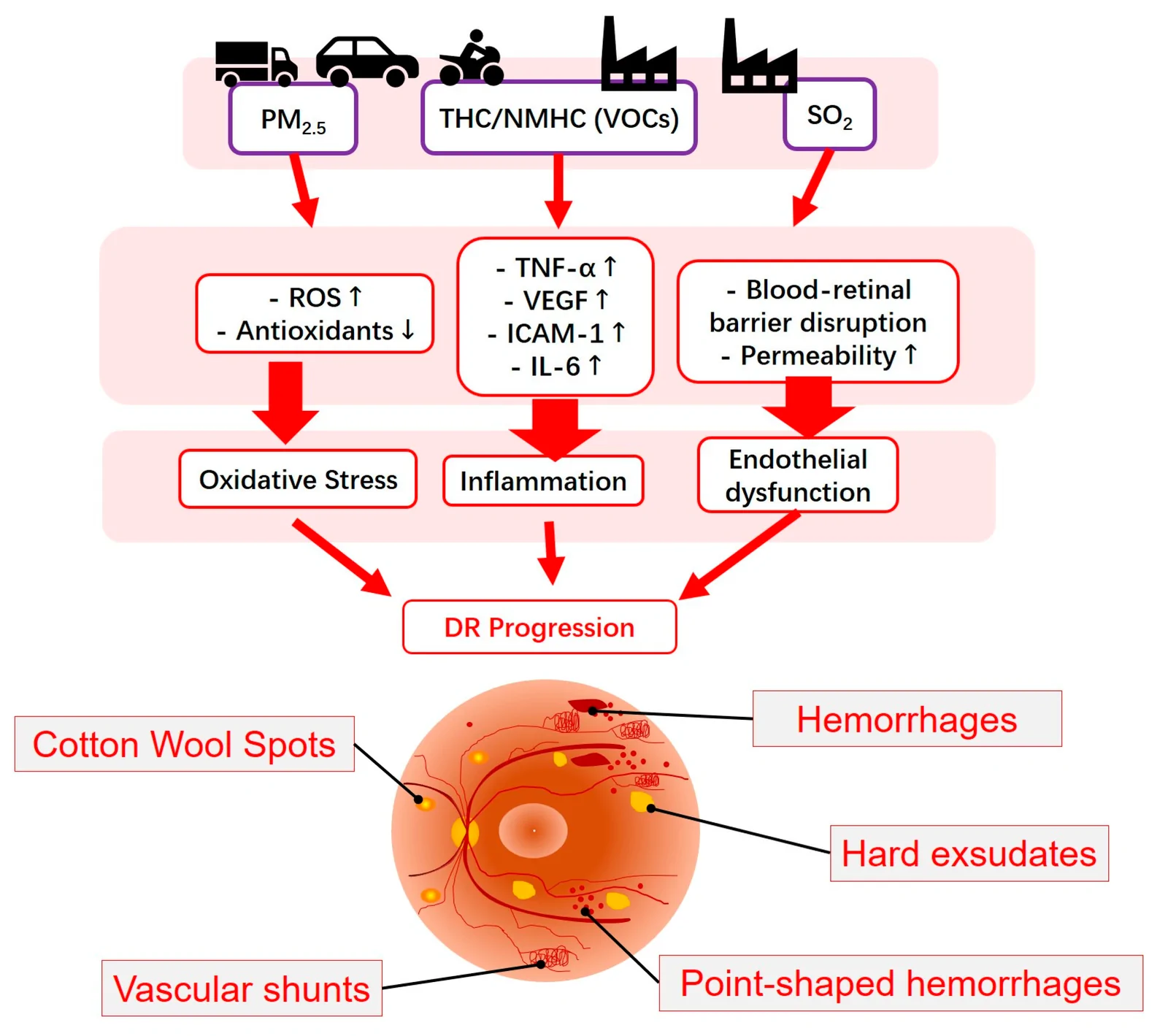
Environmental pollutants (THC/NMHC, PM_2.5_, SO_2_) drive the microvascular damage and proliferative progression of diabetic retinopathy by inducing oxidative stress (ROS ↑, antioxidant enzymes ↓) in the retina, intensifying inflammatory responses (TNF-α, IL-6, VEGF, ICAM-1), and disrupting the blood–retinal barrier, leading to endothelial dysfunction (increased permeability).

**Figure 8:**
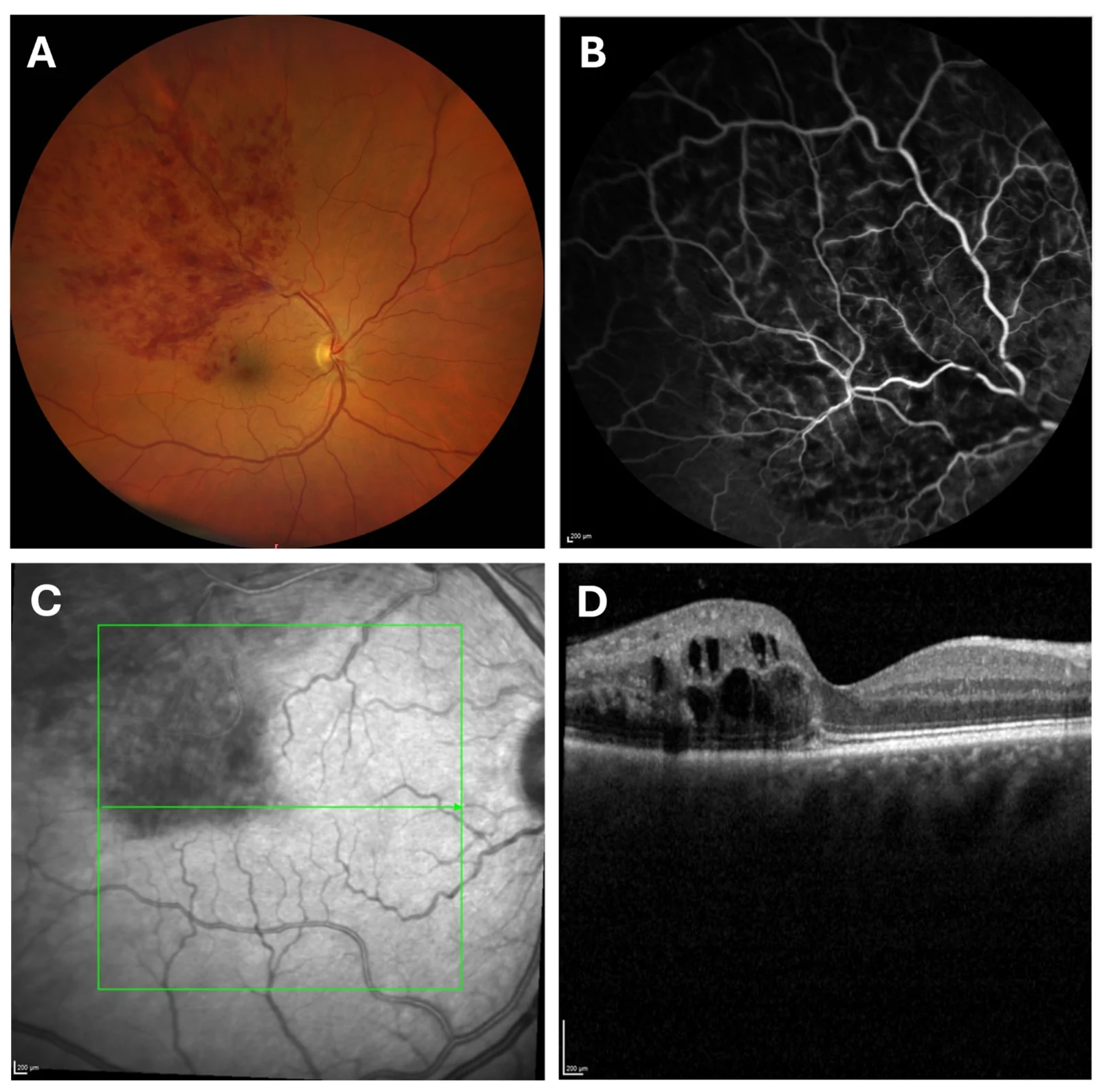
Fundus photograph of a 61-year-old patient with branch retinal vein occlusion with retinal hemorrhages and venous congestion in the affected venous drainage area of the temporal superior retinal vein (**A**). Fluorescein angiography reveals delayed venous filling and capillary non-perfusion in the affected retinal sector (**B**). Optical coherence tomography (OCT) shows subsequent cystoid macular edema (**C**,**D**). The green rectangular box (**C**) indicates the area selected for OCT analysis. The green line within the box represents the segmentation line reflecting the scan position of the scan presented in (**D**).

**Figure 9:**
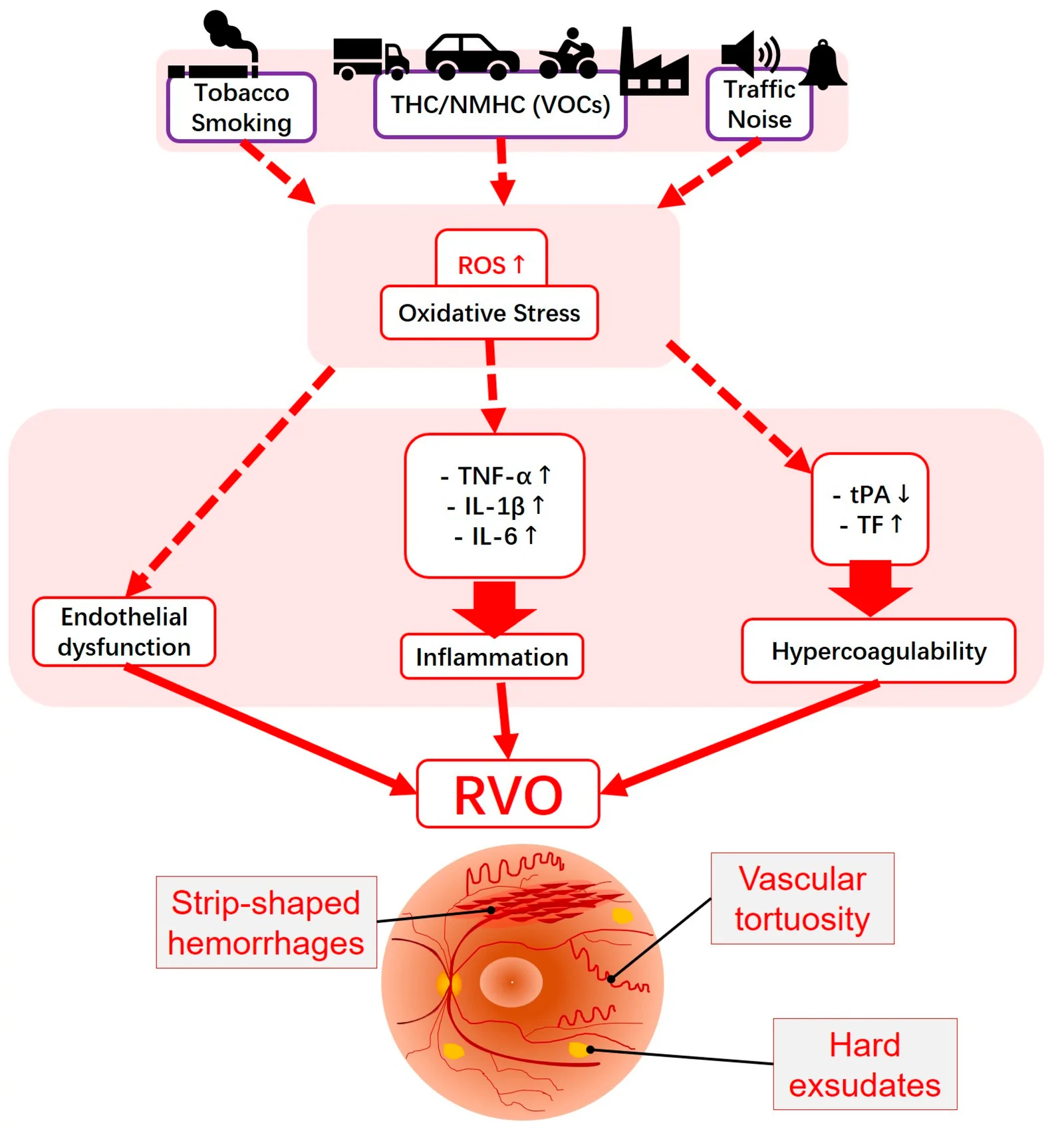
Environmental pollutants (such as THC/NMHC and other volatile organic compounds, traffic noise, and smoking) can induce an increase in reactive oxygen species (ROS ↑), leading to endothelial dysfunction of the blood–retinal barrier, activating inflammatory factors (IL-1β, TNF-α, IL-6), and promoting a procoagulant state (upregulation of tissue factor and downregulation of tPA), all of which jointly contribute to the occurrence and development of retinal vein occlusion (RVO).

**Figure 10:**
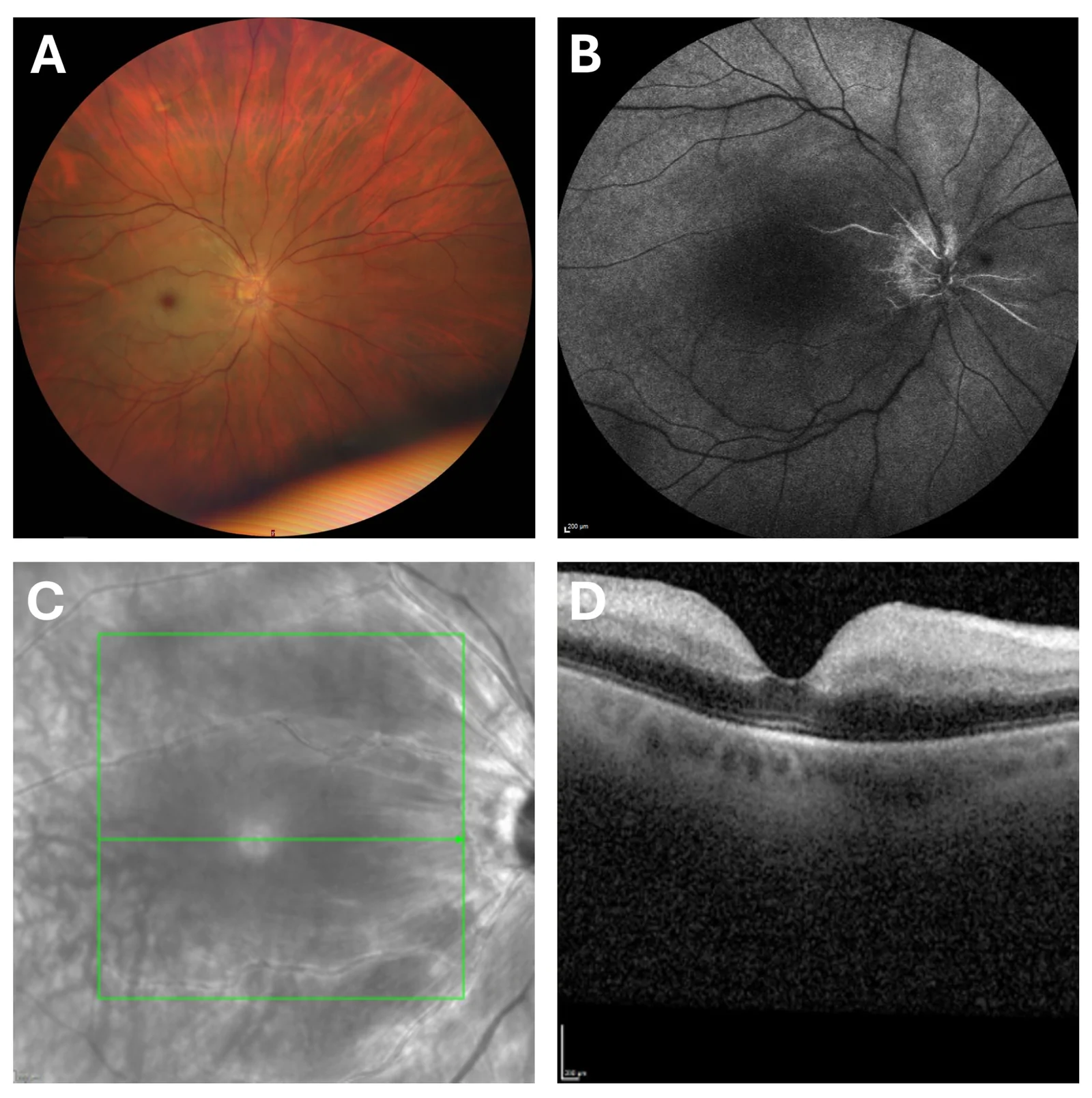
Central retinal artery occlusion in the right eye of a 65-year-old patient. Fundus photograph with characteristic cherry-red spot at the fovea (**A**). Fluorescein angiography demonstrates almost completely absent filling of the central retinal artery and its branches with subsequent retinal non-perfusion (**B**). Optical coherence tomography (OCT) shows hyperreflectivity and thickening of inner retinal layers as a consequence of retinal ischemia (**C**,**D**). The green rectangular box (**C**) indicates the area selected for OCT analysis. The green line within the box represents the segmentation line reflecting the scan position of the scan presented in (**D**). Scale: 200 μm.

**Figure 11:**
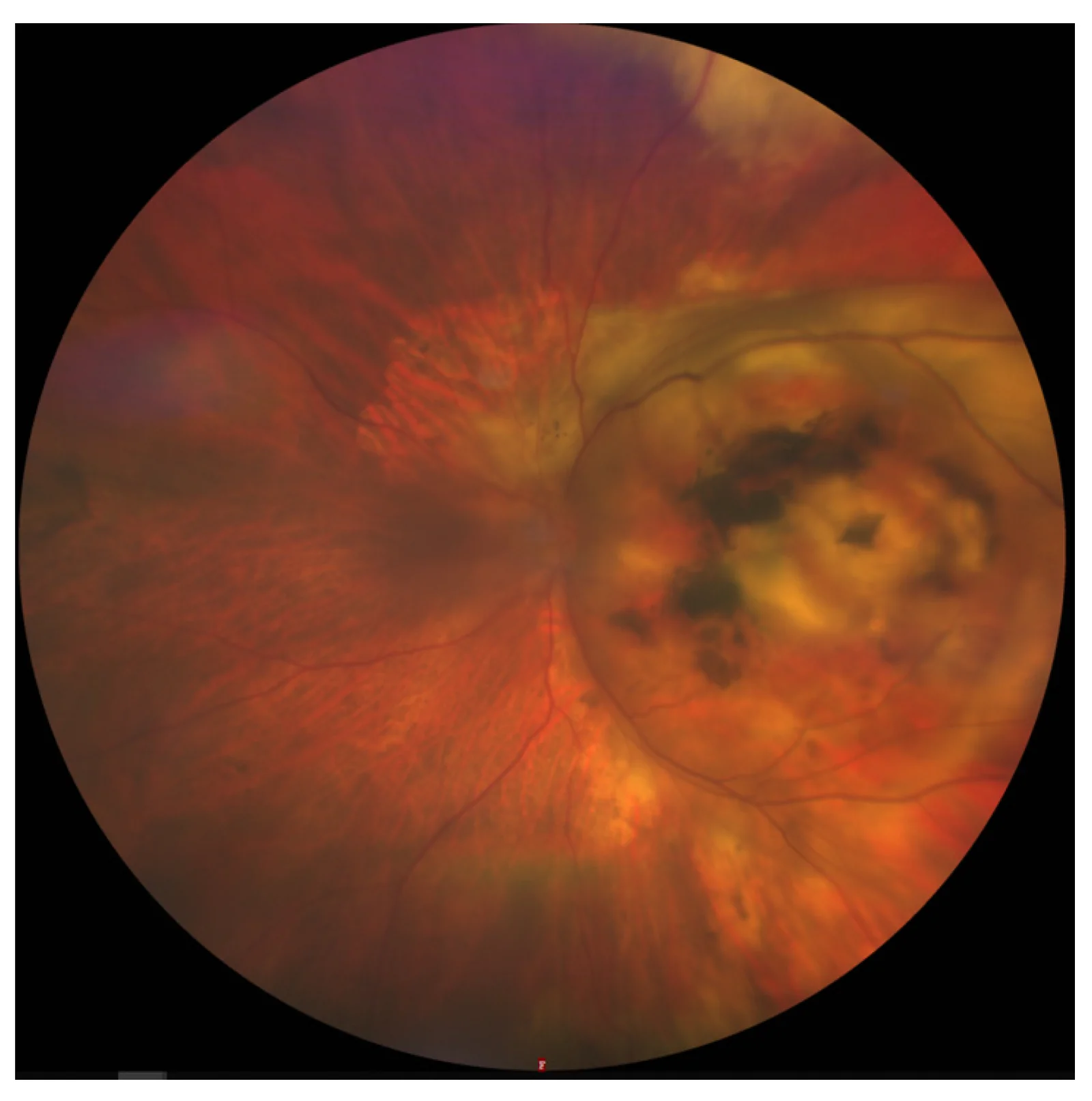
Fundus photograph of a 84-year-old patient with age-related macular degeneration and temporal retinal detachment involving the macula.

## Data Availability

No new data were created or analyzed in this study. Data sharing is not applicable to this article.

## References

[1] Piepoli M., Weidinger F., Aboyans V., Abreu A., Andersen Z.J., Baneras J., Carugno M., Cerbai E., Castelvecchio S., Daiber A. (2026). Environmental risk factors and cardiovascular health. Eur. Heart J..

[2] Schraufnagel D.E., Balmes J.R., Cowl C.T., De Matteis S., Jung S.H., Mortimer K., Perez-Padilla R., Rice M.B., Riojas-Rodriguez H., Sood A. (2019). Air Pollution and Noncommunicable Diseases: A Review by the Forum of International Respiratory Societies’ Environmental Committee, Part 2: Air Pollution and Organ Systems. Chest.

[3] Sicard P., Agathokleous E., Anenberg S.C., De Marco A., Paoletti E., Calatayud V. (2023). Trends in urban air pollution over the last two decades: A global perspective. Sci. Total Environ..

[4] Gupta P., Muthukumar A. (2018). Minor to chronic eye disorders due to environmental pollution: A review. J. Ocul. Infect. Inflamm..

[5] Jung S.J., Mehta J.S., Tong L. (2018). Effects of environment pollution on the ocular surface. Ocul. Surf..

[6] Van Kempen E., Casas M., Pershagen G., Foraster M. (2018). WHO Environmental Noise Guidelines for the European Region: A Systematic Review on Environmental Noise and Cardiovascular and Metabolic Effects: A Summary. Int. J. Environ. Res. Public Health.

[7] Hoek G., Krishnan R.M., Beelen R., Peters A., Ostro B., Brunekreef B., Kaufman J.D. (2013). Long-term air pollution exposure and cardio- respiratory mortality: A review. Environ. Health.

[8] Karimi B., Samadi S. (2024). Long-term exposure to air pollution on cardio-respiratory, and lung cancer mortality: A systematic review and meta-analysis. J. Environ. Health Sci. Eng..

[9] Zhao J., Gong F., Yang Q., Yang R., Yan Z., Xi Z., Li K., Lai W., Shi Y., Lin B. (2024). Exercise in ozone-polluted air evokes pathological cardiac hypertrophy via up-regulation of nuclear lncRNA EYA4-au1 and recruiting Med11 to activating EYA4/p27kip1/CK2α/HDAC2 cascade. Ecotoxicol. Environ. Saf..

[10] Tian Y., Ma Y., Xu R., Wu Y., Li S., Hu Y., Guo Y. (2024). Landscape fire PM_2.5_ and hospital admissions for cause-specific cardiovascular disease in urban China. Nat. Commun..

[11] Huang X., Wang Z., Lei F., Liu W., Lin L., Sun T., Cao Y., Zhang X., Cai J., Li H. (2024). Association of urban environments with Atherosclerotic cardiovascular disease: A prospective cohort study in the UK Biobank. Environ. Int..

[12] Hahad O., Rajagopalan S., Lelieveld J., Sorensen M., Frenis K., Daiber A., Basner M., Nieuwenhuijsen M., Brook R.D., Munzel T. (2023). Noise and Air Pollution as Risk Factors for Hypertension: Part I-Epidemiology. Hypertension.

[13] Hahad O., Rajagopalan S., Lelieveld J., Sorensen M., Kuntic M., Daiber A., Basner M., Nieuwenhuijsen M., Brook R.D., Munzel T. (2023). Noise and Air Pollution as Risk Factors for Hypertension: Part II-Pathophysiologic Insight. Hypertension.

[14] Qin S.J., Zeng Q.G., Zeng H.X., Li S.P., Andersson J., Zhao B., Oudin A., Kanninen K.M., Jalava P., Jin N.X. (2024). Neurotoxicity of fine and ultrafine particulate matter: A comprehensive review using a toxicity pathway-oriented adverse outcome pathway framework. Sci. Total Environ..

[15] Meng L., Ouyang Z., Chen Y., Huang C., Yu Y., Fan R. (2024). Low-dose BPA-induced neuronal energy metabolism dysfunction and apoptosis mediated by PINK1/parkin mitophagy pathway in juvenile rats. Sci. Total Environ..

[16] Dorsey E.R., De Miranda B.R., Horsager J., Borghammer P. (2024). The Body, the Brain, the Environment, and Parkinson’s Disease. J. Park. Dis..

[17] Liu S., He Y., Yin J., Zhu Q., Liao C., Jiang G. (2024). Neurotoxicities induced by micro/nanoplastics: A review focusing on the risks of neurological diseases. J. Hazard. Mater..

[18] Zhang Y., Meliefste K., Hu W., Portengen L., Rothman N., Reiss B., Li J., Xu J., Ning B., Liu D. (2024). Expanded PAH analysis of household air pollution in a rural region of China with high lung cancer incidence. Environ. Pollut..

[19] Huang H., Hou J., Yu C., Wei F., Xi B. (2024). Microplastics exacerbate tissue damage and promote carcinogenesis following liver infection in mice. Ecotoxicol. Environ. Saf..

[20] Steinmetz J.D., Bourne R.R., Briant P.S., Flaxman S.R., Taylor H.R., Jonas J.B., Abdoli A.A., Abrha W.A., Abualhasan A., Abu-Gharbieh E.G. (2021). Causes of blindness and vision impairment in 2020 and trends over 30 years, and prevalence of avoidable blindness in relation to VISION 2020: The Right to Sight: An analysis for the Global Burden of Disease Study. Lancet Glob. Health.

[21] Solebo A.L., Teoh L., Rahi J. (2017). Epidemiology of blindness in children. Arch. Dis. Child..

[22] Bourne R., Steinmetz J.D., Flaxman S., Briant P.S., Taylor H.R., Resnikoff S., Casson R.J., Abdoli A., Abu-Gharbieh E., Afshin A. (2021). Trends in prevalence of blindness and distance and near vision impairment over 30 years: An analysis for the Global Burden of Disease Study. Lancet Glob. Health.

[23] Marques A.P., Ramke J., Cairns J., Butt T., Zhang J.H., Muirhead D., Jones I., Tong B.A.M.A., Swenor B.K., Faal H. (2021). Global economic productivity losses from vision impairment and blindness. eClinicalMedicine.

[24] Domènech E.B., Marfany G. (2020). The Relevance of Oxidative Stress in the Pathogenesis and Therapy of Retinal Dystrophies. Antioxidants.

[25] Li Y., Zhao T., Li J., Xia M., Li Y., Wang X., Liu C., Zheng T., Chen R., Kan D. (2022). Oxidative Stress and 4-hydroxy-2-nonenal (4-HNE): Implications in the Pathogenesis and Treatment of Aging-related Diseases. J. Immunol. Res..

[26] Li J., Hu Y., Liu L., Wang Q., Zeng J., Chen C. (2020). PM2.5 exposure perturbs lung microbiome and its metabolic profile in mice. Sci. Total Environ..

[27] Liu G., Li Y., Zhou J., Xu J., Yang B. (2022). PM2.5 deregulated microRNA and inflammatory microenvironment in lung injury. Environ. Toxicol. Pharmacol..

[28] Han H., Oh E.Y., Lee J.H., Park J.W., Park H.J. (2021). Effects of Particulate Matter 10 Inhalation on Lung Tissue RNA expression in a Murine Model. Tuberc. Respir. Dis..

[29] Xie W., You J., Zhi C., Li L. (2021). The toxicity of ambient fine particulate matter (PM2.5) to vascular endothelial cells. J. Appl. Toxicol..

[30] Kuntic M., Kuntic I., Krishnankutty R., Gericke A., Oelze M., Junglas T., Jimenez M.T.B., Stamm P., Nandudu M., Hahad O. (2023). Co-exposure to urban particulate matter and aircraft noise adversely impacts the cerebro-pulmonary-cardiovascular axis in mice. Redox Biol..

[31] Chen C.T., Lin Z.H., Lin T.Y., Lin Y.A., Yeh H., Hua W.J., Tsai C.Y. (2025). PM2.5 exposure induces transcriptomic changes in ARPE-19 cells with activation of TGFβ-mediated signaling pathways: A next-generation sequencing approach. J. Chin. Med. Assoc..

[32] Lin H.W., Shen T.J., Chen P.Y., Chen T.C., Yeh J.H., Tsou S.C., Lai C.Y., Chen C.H., Chang Y.Y. (2022). Particulate matter 2.5 exposure induces epithelial-mesenchymal transition via PI3K/AKT/mTOR pathway in human retinal pigment epithelial ARPE-19 cells. Biochem Biophys. Res. Commun..

[33] Choi J.Y., Bae J.E., Kim J.B., Jo D.S., Park N.Y., Kim Y.H., Lee H.J., Kim S.H., Kim S.H., Jeon H.B. (2021). 2-IPMA Ameliorates PM2.5-Induced Inflammation by Promoting Primary Ciliogenesis in RPE Cells. Molecules.

[34] Kim J.Y., Kim J.H., Kim Y.D., Seo J.H. (2022). Ultrafine Diesel Exhaust Particles Induce Apoptosis of Oligodendrocytes by Increasing Intracellular Reactive Oxygen Species through NADPH Oxidase Activation. Antioxidants.

[35] Chronopoulos P., Manicam C., Zadeh J.K., Laspas P., Unkrig J.C., Göbel M.L., Musayeva A., Pfeiffer N., Oelze M., Daiber A. (2023). Effects of Resveratrol on Vascular Function in Retinal Ischemia-Reperfusion Injury. Antioxidants.

[36] Kim J.Y., Lee E.Y., Kim J.H., Seo E.J., Eom S.Y., Seo J.H. (2025). Diesel exhaust particles disrupt blood-retina barrier integrity via TLR2 and TLR4 activation. BMB Rep..

[37] Lee H., Hwang-Bo H., Ji S.Y., Kim M.Y., Kim S.Y., Park C., Hong S.H., Kim G.Y., Song K.S., Hyun J.W. (2020). Diesel particulate matter2.5 promotes epithelial-mesenchymal transition of human retinal pigment epithelial cells via generation of reactive oxygen species. Environ. Pollut..

[38] Li H., Yan Y., Yan H., Luo Y., Wang H., Zhao L., Zang R., Zeng Q., Cui T., Zhou J. (2025). Chronic low-dose cadmium exposure induces neurodevelopmental impairment in hESC-derived retinal organoids. Ecotoxicol. Environ. Saf..

[39] Kalariya N.M., Wills N.K., Ramana K.V., Srivastava S.K., van Kuijk F.J. (2009). Cadmium-induced apoptotic death of human retinal pigment epithelial cells is mediated by MAPK pathway. Exp. Eye Res..

[40] Zhang L., Xia Q., Zhou Y., Li J. (2019). Endoplasmic reticulum stress and autophagy contribute to cadmium-induced cytotoxicity in retinal pigment epithelial cells. Toxicol. Lett..

[41] Demirtaş S., Gökdemir G.S., Şeker U. (2026). Cadmium-induced systemic inflammation and retinal degeneration: Histopathological and cytokine analysis in a rat model. Cutan. Ocul. Toxicol..

[42] Wills N.K., Kalariya N., Ramanujam V.M.S., Lewis J.R., Abdollahi S.H., Husain A., van Kuijk F.J. (2009). Human retinal cadmium accumulation as a factor in the etiology of age-related macular degeneration. Exp. Eye Res..

[43] Sharma A., Neekhra A., Gramajo A.L., Patil J., Chwa M., Kuppermann B.D., Kenney M.C. (2008). Effects of Benzo(e)Pyrene, a toxic component of cigarette smoke, on human retinal pigment epithelial cells in vitro. Investig. Ophthalmol. Vis. Sci..

[44] Kasikci M., Sen S. (2024). Resveratrol and quercetin protect from Benzo(a)pyrene-induced autophagy in retinal pigment epithelial cells. Int. Ophthalmol..

[45] Patil A.J., Gramajo A.L., Sharma A., Chwa M., Seigel G.M., Kuppermann B.D., Kenney M.C. (2009). Effects of benzo(e)pyrene on the retinal neurosensory cells and human microvascular endothelial cells in vitro. Curr. Eye Res..

[46] Kamiya T., Nagaoka T., Omae T., Ono S., Otani S., Yoshida A. (2017). Benzo(e)pyrene Inhibits Endothelium-Dependent NO-Mediated Dilation of Retinal Arterioles via Superoxide Production and Endoplasmic Reticulum Stress. Investig. Ophthalmol. Vis. Sci..

[47] Peretz A., Sullivan J.H., Leotta D.F., Trenga C.A., Sands F.N., Allen J., Carlsten C., Wilkinson C.W., Gill E.A., Kaufman J.D. (2008). Diesel exhaust inhalation elicits acute vasoconstriction in vivo. Environ. Health Perspect..

[48] Matsumoto G., Nakagawa N.K., de Paula Vieira R., Mauad T., da Silva L.F.F., de André C.D.S., Carvalho-Oliveira R., Saldiva P.H.N., Garcia M.L.B. (2010). The time course of vasoconstriction and endothelin receptor A expression in pulmonary arterioles of mice continuously exposed to ambient urban levels of air pollution. Environ. Res..

[49] Khalil-Manesh F., Gonick H.C., Weiler E.W., Prins B., Weber M.A., Purdy R.E. (1993). Lead-induced hypertension: Possible role of endothelial factors. Am. J. Hypertens..

[50] Omanwar S., Fahim M. (2015). Mercury Exposure and Endothelial Dysfunction: An Interplay Between Nitric Oxide and Oxidative Stress. Int. J. Toxicol..

[51] Lee M.Y., Lee Y.H., Lim K.M., Chung S.M., Bae O.N., Kim H., Lee C.R., Park J.D., Chung J.H. (2005). Inorganic arsenite potentiates vasoconstriction through calcium sensitization in vascular smooth muscle. Environ. Health Perspect..

[52] Yuan G., Liu Y., Wang Z., Wang X., Han Z., Yan X., Meng A. (2022). PM2.5 activated NLRP3 inflammasome and IL-1β release in MH-S cells by facilitating autophagy via activating Wnt5a. Int. J. Immunopathol. Pharmacol..

[53] Park H.S., Oh M.K., Lee J.W., Chae D.H., Joo H., Kang J.Y., An H.B., Yu A., Park J.H., Yoo H.M. (2022). Diesel Exhaust Particles Impair Therapeutic Effect of Human Wharton’s Jelly-Derived Mesenchymal Stem Cells against Experimental Colitis through ROS/ERK/cFos Signaling Pathway. Int. J. Stem Cells.

[54] Xia C., Capozzi S.L., Romanak K.A., Lehman D.C., Dove A., Richardson V., Greenberg T., McGoldrick D., Venier M. (2024). The Ins and Outs of Per- and Polyfluoroalkyl Substances in the Great Lakes: The Role of Atmospheric Deposition. Environ. Sci. Technol..

[55] Dimitrakopoulou M.-E., Karvounis M., Marinos G., Theodorakopoulou Z., Aloizou E., Petsangourakis G., Papakonstantinou M., Stoitsis G. (2024). Comprehensive analysis of PFAS presence from environment to plate. npj Sci. Food.

[56] Wu L.Y., Zhang J.L., Zeeshan M., Zhou Y., Zhang Y.T., He W.T., Jin N., Dai Y., Chi W., Ou Z. (2024). Caspase-8 promotes NLRP3 inflammasome activation mediates eye development defects in zebrafish larvae exposed to perfulorooctane sulfonate (PFOS). Environ. Pollut..

[57] Kim D.H., Lee H., Kim M.Y., Hwangbo H., Ji S.Y., Bang E., Hong S.H., Kim G.Y., Leem S.H., Ryu D. (2025). Particulate matter 2.5 stimulates pyroptosis and necroptosis via the p38 MAPK/Akt/NF-κB signaling pathway in human corneal epithelial cells. Toxicology.

[58] Kong H., Zhao H., Chen T., Song Y., Cui Y. (2022). Targeted P2X7/NLRP3 signaling pathway against inflammation, apoptosis, and pyroptosis of retinal endothelial cells in diabetic retinopathy. Cell Death Dis..

[59] Sun Y., Li F., Liu Y., Qiao D., Yao X., Liu G.S., Li D., Xiao C., Wang T., Chi W. (2024). Targeting inflammasomes and pyroptosis in retinal diseases-molecular mechanisms and future perspectives. Prog. Retin. Eye Res..

[60] Gu Y., Sheng F., Gao M., Zhang L., Hao S., Chen S., Chen R., Xu Y., Wu D., Han Y. (2023). Acute and continuous exposure of airborne fine particulate matter (PM_2.5_): Diverse outer blood-retinal barrier damages and disease susceptibilities. Part. Fibre Toxicol..

[61] Gu Y., Hao S., Liu K., Gao M., Lu B., Sheng F., Zhang L., Xu Y., Wu D., Han Y. (2022). Airborne fine particulate matter (PM_2.5_) damages the inner blood-retinal barrier by inducing inflammation and ferroptosis in retinal vascular endothelial cells. Sci. Total Environ..

[62] Zhao L., Wu P., Lu J., He Y., Shu Q., Pan F., Xie H., Wang X., Ju H., Du Y. (2024). Cigarette smoke extract induces ferroptosis in human retinal pigment epithelial cells. Heliyon.

[63] Yang B., Yang K., Chen Y., Li Q., Chen J., Li S., Wu Y. (2025). Exposure of A2E to blue light promotes ferroptosis in the retinal pigment epithelium. Cell. Mol. Biol. Lett..

[64] Xiang W., Li L., Zhao Q., Zeng Y., Shi J., Chen Z., Gao G., Lai K. (2024). PEDF protects retinal pigment epithelium from ferroptosis and ameliorates dry AMD-like pathology in a murine model. Geroscience.

[65] Lee H., Kim D.H., Kim J.H., Park S.K., Jeong J.W., Kim M.Y., Hong S.H., Song K.S., Kim G.Y., Hyun J.W. (2021). Urban Aerosol Particulate Matter Promotes Necrosis and Autophagy via Reactive Oxygen Species-Mediated Cellular Disorders that are Accompanied by Cell Cycle Arrest in Retinal Pigment Epithelial Cells. Antioxidants.

[66] Villarejo-Zori B., Zapata-Muñoz J., Sierra-Filardi E., Ramírez-Pardo I., Montava-Garriga L., Ganley I.G., Boya P. (2025). Deferiprone protects against photoreceptor degeneration by inhibiting parthanatos. Cell Death Dis..

[67] Zinflou C., Rochette P.J. (2023). Indenopyrene and Blue-Light Co-Exposure Impairs the Tightly Controlled Activation of Xenobiotic Metabolism in Retinal Pigment Epithelial Cells: A Mechanism for Synergistic Toxicity. Int. J. Mol. Sci..

[68] Ji J., Xiong C., Yang H., Jiang Z., Zhang Y., Wang X., Yu T., Li Q., Zhu S., Zhou Y. (2024). The aryl hydrocarbon receptor: A crucial mediator in ocular disease pathogenesis and therapeutic target. Exp. Eye Res..

[69] Yang T.M., Fang T.C., Lee Y.C., Lee C.C., Chan Y.J., Fitriana I., Cheng Y.W., Li C.H. (2025). Aryl Hydrocarbon Receptor Deficiency Upregulates Intercellular Adhesion Molecule 1 in Retinal Pigment Epithelial Cells and Contributes to Retinal Inflammation. Lab. Investig..

[70] Resnikoff S., Pascolini D., Etya’ale D., Kocur I., Pararajasegaram R., Pokharel G.P., Mariotti S.P. (2004). Global data on visual impairment in the year 2002. Bull. World Health Organ..

[71] Wong W.L., Su X., Li X., Cheung C.M.G., Klein R., Cheng C.Y., Wong T.Y. (2014). Global prevalence of age-related macular degeneration and disease burden projection for 2020 and 2040: A systematic review and meta-analysis. Lancet Glob. Health.

[72] Paudel N., Brady L., Stratieva P., Galvin O., Lui B., Van den Brande I., Malkowski J.P., Rebeira M., MacAllister S., O’Riordan T. (2024). Economic Burden of Late-Stage Age-Related Macular Degeneration in Bulgaria, Germany, and the US. JAMA Ophthalmol..

[73] Ma H.H., Liutkeviciene R. (2021). Age-Related Macular Degeneration: What Do We Know So Far?. Acta Medica Litu..

[74] Sheth J.U., Stewart M.W., Narayanan R., Anantharaman G., Chandran K., Lai T.Y.Y., Chakravarthy U., Das T. (2025). Macular neovascularization. Surv. Ophthalmol..

[75] Spooner K., Broadhead G., Fraser-Bell S., Hong T., Wong J.G., Chang A.A. (2025). Real-World 10-Year Outcomes of Anti-VEGF Therapy for Neovascular Age-Related Macular Degeneration: A Meta-Analysis. Clin. Exp. Ophthalmol..

[76] Han J.H., Amri C., Lee H., Hur J. (2024). Pathological Mechanisms of Particulate Matter-Mediated Ocular Disorders: A Review. Int. J. Mol. Sci..

[77] Brodzka S., Baszynski J., Rektor K., Holderna-Bona K., Stanek E., Kurhaluk N., Tkaczenko H., Malukiewicz G., Wozniak A., Kaminski P. (2024). Immunogenetic and Environmental Factors in Age-Related Macular Disease. Int. J. Mol. Sci..

[78] Chang K.H., Hsu P.Y., Lin C.J., Lin C.L., Juo S.H., Liang C.L. (2019). Traffic-related air pollutants increase the risk for age-related macular degeneration. J. Investig. Med..

[79] Zhang H.W., Lin C.W., Kok V.C., Tseng C.H., Lin Y.P., Li T.C., Sung F.C., Wen C.P., Hsiung C.A., Hsu C.Y. (2019). Incidence of retinal vein occlusion with long-term exposure to ambient air pollution. PLoS ONE.

[80] Grant A., Leung G., Freeman E.E. (2022). Ambient Air Pollution and Age-Related Eye Disease: A Systematic Review and Meta-Analysis. Investig. Ophthalmol. Vis. Sci..

[81] Chua S.Y.L., Warwick A., Peto T., Balaskas K., Moore A.T., Reisman C., Desai P., Lotery A.J., Dhillon B., Khaw P.T. (2022). Association of ambient air pollution with age-related macular degeneration and retinal thickness in UK Biobank. Br. J. Ophthalmol..

[82] Grant A., Leung G., Aubin M.J., Kergoat M.J., Li G., Freeman E.E. (2021). Fine Particulate Matter and Age-Related Eye Disease: The Canadian Longitudinal Study on Aging. Investig. Ophthalmol. Vis. Sci..

[83] He J., Liu Y., Zhang A., Liu Q., Yang X., Sun N., Yao B., Liang F., Yan X., Liu Y. (2023). Joint effects of meteorological factors and PM_2.5_ on age-related macular degeneration: A national cross-sectional study in China. Environ. Health Prev. Med..

[84] Jia H., Guo Y., Luo H., Meng X., Zhang L., Yu K., Zheng X., Sun Y., Hu W., Wu Z. (2024). Association of long-term ozone air pollution and age-related macular degeneration in older Chinese population. Sci. Total Environ..

[85] Chakravarthy U., Augood C., Bentham G.C., de Jong P.T., Rahu M., Seland J., Soubrane G., Tomazzoli L., Topouzis F., Vingerling J.R. (2007). Cigarette smoking and age-related macular degeneration in the EUREYE Study. Ophthalmology.

[86] Smith W., Mitchell P., Leeder S.R. (1996). Smoking and age-related maculopathy: The Blue Mountains Eye Study. Arch. Ophthalmol..

[87] Bertram K.M., Baglole C.J., Phipps R.P., Libby R.T. (2009). Molecular regulation of cigarette smoke induced-oxidative stress in human retinal pigment epithelial cells: Implications for age-related macular degeneration. Am. J. Physiol.-Cell Physiol..

[88] Smit-McBride Z., Nguyen J., Elliott G.W., Wang Z., McBride R.A., Nguyen A.T., Oltjen S.L., Yiu G., Thomasy S.M., Pinkerton K.E. (2018). Effects of aging and environmental tobacco smoke exposure on ocular and plasma circulatory microRNAs in the Rhesus macaque. Mol. Vis..

[89] Tien P.T., Lin H.J., Tsai Y.Y., Lim Y.P., Chen C.S., Chang C.Y., Lin C.J., Chen J.J., Wu S.M., Huang Y.J. (2020). Perfluorooctanoic acid in indoor particulate matter triggers oxidative stress and inflammation in corneal and retinal cells. Sci. Rep..

[90] Fleckenstein M., Keenan T.D.L., Guymer R.H., Chakravarthy U., Schmitz-Valckenberg S., Klaver C.C., Wong W.T., Chew E.Y. (2021). Age-related macular degeneration. Nat. Rev. Dis. Primers.

[91] Kang Y.J., Tan H.Y., Lee C.Y., Cho H. (2021). An Air Particulate Pollutant Induces Neuroinflammation and Neurodegeneration in Human Brain Models. Adv. Sci..

[92] Zeng Y., Li M., Zou T., Chen X., Li Q., Li Y., Ge L., Chen S., Xu H. (2021). The Impact of Particulate Matter (PM2.5) on Human Retinal Development in hESC-Derived Retinal Organoids. Front. Cell Dev. Biol..

[93] Chua S.Y.L., Khawaja A.P., Dick A.D., Morgan J., Dhillon B., Lotery A.J., Strouthidis N.G., Reisman C., Peto T., Khaw P.T. (2020). Ambient Air Pollution Associations with Retinal Morphology in the UK Biobank. Investig. Ophthalmol. Vis. Sci..

[94] Datta M., Bessen B.M., Patel M.J., Hackam A.S. (2026). Cellular responses of human Muller glia exposed to test dust pollution. Environ. Pollut..

[95] Kim S.H., Kim Y.K., Shin Y.I., Kang G., Kim S.P., Lee H., Hong I.H., Chang I.B., Hong S.B., Yoon H.J. (2024). Nighttime Outdoor Artificial Light and Risk of Age-Related Macular Degeneration. JAMA Netw. Open.

[96] Behar-Cohen F., Martinsons C., Vienot F., Zissis G., Barlier-Salsi A., Cesarini J.P., Enouf O., Garcia M., Picaud S., Attia D. (2011). Light-emitting diodes (LED) for domestic lighting: Any risks for the eye?. Prog. Retin. Eye Res..

[97] Zhu S., Li X., Dang B., Wu F., Gou K., Wang C., Lin C. (2022). Hydrogen sulfide protects retina from blue light-induced photodamage and degeneration via inhibiting ROS-mediated ER stress-CHOP apoptosis signal. Redox Rep..

[98] International Commission on Non-Ionizing Radiation Protection (ICNIRP) (2013). ICNIRP Guidelines on Limits of Exposure to Incoherent Visible and Infrared Radiation. Health Phys..

[99] O’Hagan J.B., Khazova M., Price L.L.A. (2016). Low-energy light bulbs, computers, tablets and the blue light hazard. Eye.

[100] Jarrett S.G., Boulton M.E. (2012). Consequences of oxidative stress in age-related macular degeneration. Mol. Asp. Med..

[101] Totsuka K., Ueta T., Uchida T., Roggia M.F., Nakagawa S., Vavvas D.G., Honjo M., Aihara M. (2019). Oxidative stress induces ferroptotic cell death in retinal pigment epithelial cells. Exp. Eye Res..

[102] Arnault E., Barrau C., Nanteau C., Gondouin P., Bigot K., Vienot F., Gutman E., Fontaine V., Villette T., Cohen-Tannoudji D. (2013). Phototoxic action spectrum on a retinal pigment epithelium model of age-related macular degeneration exposed to sunlight normalized conditions. PLoS ONE.

[103] Kuse Y., Ogawa K., Tsuruma K., Shimazawa M., Hara H. (2014). Damage of photoreceptor-derived cells in culture induced by light emitting diode-derived blue light. Sci. Rep..

[104] Jaadane I., Villalpando Rodriguez G., Boulenguez P., Carre S., Dassieni I., Lebon C., Chahory S., Behar-Cohen F., Martinsons C., Torriglia A. (2020). Retinal phototoxicity and the evaluation of the blue light hazard of a new solid-state lighting technology. Sci. Rep..

[105] Fietz A., Corsi F., Hurst J., Schnichels S. (2023). Blue Light Damage and p53: Unravelling the Role of p53 in Oxidative-Stress-Induced Retinal Apoptosis. Antioxidants.

[106] Tao J.X., Zhou W.C., Zhu X.G. (2019). Mitochondria as Potential Targets and Initiators of the Blue Light Hazard to the Retina. Oxidative Med. Cell. Longev..

[107] Alaimo A., Liñares G.G., Bujjamer J.M., Gorojod R.M., Alcon S.P., Martínez J.H., Baldessari A., Grecco H.E., Kotler M.L. (2019). Toxicity of blue led light and A2E is associated to mitochondrial dynamics impairment in ARPE-19 cells: Implications for age-related macular degeneration. Arch. Toxicol..

[108] Li X., Zhu S., Qi F. (2023). Blue light pollution causes retinal damage and degeneration by inducing ferroptosis. J. Photochem. Photobiol. B Biol..

[109] Pipis A., Touliou E., Pillunat L.E., Augustin A.J. (2015). Effect of the blue filter intraocular lens on the progression of geographic atrophy. Eur. J. Ophthalmol..

[110] Nagai H., Hirano Y., Yasukawa T., Morita H., Nozaki M., Wolf-Schnurrbusch U., Wolf S., Ogura Y. (2015). Prevention of increased abnormal fundus autofluorescence with blue light-filtering intraocular lenses. J. Cataract Refract. Surg..

[111] Lee J.S., Li P.R., Hou C.H., Lin K.K., Kuo C.F., See L.C. (2022). Effect of Blue Light-Filtering Intraocular Lenses on Age-Related Macular Degeneration: A Nationwide Cohort Study with 10-Year Follow-up. Am. J. Ophthalmol..

[112] Kim J.Y., Kim S., Cho J., Lee J.S., Lee C.S., Byeon S.H., Kim S.S., Lee S.W., Kim Y.J. (2025). Exudative AMD Risk Following Blue Light-Filtering IOL Implantation: A Population-Based Study According to Non-Exudative AMD Status. Am. J. Ophthalmol..

[113] Hamel T., Rheault J., Simonyan D., Bourgault S., Rochette P.J. (2021). The Influence of Blue-Filtering Intraocular Lenses Implant on Exudative Age-Related Macular Degeneration: A Case-Control Study. Clin. Ophthalmol..

[114] Downie L.E., Busija L., Keller P.R. (2018). Blue-light filtering intraocular lenses (IOLs) for protecting macular health. Cochrane Database Syst. Rev..

[115] Downie L.E., Wormald R., Evans J., Virgili G., Keller P.R., Lawrenson J.G., Li T. (2019). Analysis of a Systematic Review About Blue Light-Filtering Intraocular Lenses for Retinal Protection: Understanding the Limitations of the Evidence. JAMA Ophthalmol..

[116] Vagge A., Ferro Desideri L., Del Noce C., Di Mola I., Sindaco D., Traverso C.E. (2021). Blue light filtering ophthalmic lenses: A systematic review. Semin. Ophthalmol..

[117] He J., Xiong S., Zhou W., Qiu H., Rao Y., Liu Y., Shen G., Zhao P., Chen G., Li J. (2024). Long-term polystyrene nanoparticles exposure reduces electroretinal responses and exacerbates retinal degeneration induced by light exposure. J. Hazard. Mater..

[118] Li X., Piao J., Kang B., Eom Y., Kim D.H., Song J.S. (2024). The toxic effects of polystyrene microplastic/nanoplastic particles on retinal pigment epithelial cells and retinal tissue. Environ. Sci. Pollut. Res..

[119] Yau J.W.Y., Rogers S.L., Kawasaki R., Lamoureux E.L., Kowalski J.W., Bek T., Chen S.J., Dekker J.M., Fletcher A., Grauslund J. (2012). Global prevalence and major risk factors of diabetic retinopathy. Diabetes Care.

[120] Ruta L.M., Magliano D.J., Lemesurier R., Taylor H.R., Zimmet P.Z., Shaw J.E. (2013). Prevalence of diabetic retinopathy in Type 2 diabetes in developing and developed countries. Diabet. Med..

[121] Ting D.S.W., Cheung G.C.M., Wong T.Y. (2016). Diabetic retinopathy: Global prevalence, major risk factors, screening practices and public health challenges: A review. Clin. Exp. Ophthalmol..

[122] Teo Z.L., Tham Y.C., Yu M., Chee M.L., Rim T.H., Cheung N., Bikbov M.M., Wang Y.X., Tang Y., Lu Y. (2021). Global Prevalence of Diabetic Retinopathy and Projection of Burden through 2045. Ophthalmology.

[123] Rein D.B., Zhang P., Wirth K.E., Lee P.P., Hoerger T.J., McCall N., Klein R., Tielsch J.M., Vijan S., Saaddine J. (2006). The economic burden of major adult visual disorders in the United States. Arch. Ophthalmol..

[124] Markeviciute A., Huang-Lung J., Zemaitiene R., Grzybowski A. (2023). A Review of Ambient Air Pollution as a Risk Factor for Posterior Segment Ocular Diseases. J. Clin. Med..

[125] Murata M., Noda K., Ishida S. (2020). Pathological Role of Unsaturated Aldehyde Acrolein in Diabetic Retinopathy. Front. Immunol..

[126] Antonetti D.A., Silva P.S., Stitt A.W. (2021). Current understanding of the molecular and cellular pathology of diabetic retinopathy. Nat. Rev. Endocrinol..

[127] Shukla U.V., Tripathy K. (2025). Diabetic Retinopathy.

[128] Maturi R.K., Glassman A.R., Josic K., Baker C.W., Gerstenblith A.T., Jampol L.M., Meleth A., Martin D.F., Melia M., Punjabi O.S. (2023). Four-Year Visual Outcomes in the Protocol W Randomized Trial of Intravitreous Aflibercept for Prevention of Vision-Threatening Complications of Diabetic Retinopathy. JAMA.

[129] Pan S.C., Huang C.C., Chin W.S., Chen B.Y., Chan C.C., Guo Y.L. (2020). Association between air pollution exposure and diabetic retinopathy among diabetics. Environ. Res..

[130] Shan A., Chen X., Yang X., Yao B., Liang F., Yang Z., Liu F., Chen S., Yan X., Huang J. (2021). Association between long-term exposure to fine particulate matter and diabetic retinopathy among diabetic patients: A national cross-sectional study in China. Environ. Int..

[131] Wang B., Sun Y., Zhang K., Wang Y., Tan X., Wang N., Lu Y. (2024). Long-term exposure to ambient air pollution and risk of microvascular complications among patients with type 2 diabetes: A prospective study. Int. J. Epidemiol..

[132] Tao F., Gonzalez-Flecha B., Kobzik L. (2003). Reactive oxygen species in pulmonary inflammation by ambient particulates. Free Radic. Biol. Med..

[133] Pope C.A., Bhatnagar A., McCracken J.P., Abplanalp W., Conklin D.J., O’Toole T. (2016). Exposure to Fine Particulate Air Pollution Is Associated with Endothelial Injury and Systemic Inflammation. Circ. Res..

[134] Niu J., Liberda E.N., Qu S., Guo X., Li X., Zhang J., Meng J., Yan B., Li N., Zhong M. (2013). The role of metal components in the cardiovascular effects of PM_2.5_. PLoS ONE.

[135] Zhang J., Zhang J., Zhang C., Zhang J., Gu L., Luo D., Qiu Q. (2022). Diabetic Macular Edema: Current Understanding, Molecular Mechanisms and Therapeutic Implications. Cells.

[136] Jomova K., Valko M. (2011). Advances in metal-induced oxidative stress and human disease. Toxicology.

[137] Miller M.R., Shaw C.A., Langrish J.P. (2012). From particles to patients: Oxidative stress and the cardiovascular effects of air pollution. Future Cardiol..

[138] Cugati S., Wang J.J., Rochtchina E., Mitchell P. (2006). Ten-year incidence of retinal vein occlusion in an older population: The Blue Mountains Eye Study. Arch. Ophthalmol..

[139] Knudtson M.D., Klein B.E.K., Klein R., Cruickshanks K.J., Lee K.E. (2005). Age-related eye disease, quality of life, and functional activity. Arch. Ophthalmol..

[140] Zhao J., Sastry S.M., Sperduto R.D., Chew E.Y., Remaley N.A., Yannuzzi L.A., Sorenson J.A., Seddon J.M., Gragoudas E.S., Puliafito C.A. (1993). Arteriovenous crossing patterns in branch retinal vein occlusion. Ophthalmology.

[141] Voigt A.M., Elbaz H., Bohm E.W., Wild P.S., Lackner K.J., Beutel M.E., Schmidtmann I., Tuscher O., Schattenberg J.M., Konstantinides S.V. (2025). Incidence of Retinal Vein Occlusion and Its Association with Mortality: Results from the Gutenberg Health Study. Ophthalmology.

[142] Hariprasad S.M., Holz F.G., Asche C.V., Issa A., Mora O., Keady S., Rezk M.F., Sarocco P., Simoens S. (2025). Clinical and Socioeconomic Burden of Retinal Diseases: Can Biosimilars Add Value? A Narrative Review. Ophthalmol. Ther..

[143] Lendzioszek M., Bryl A., Poppe E., Zorena K., Mrugacz M. (2024). Retinal Vein Occlusion-Background Knowledge and Foreground Knowledge Prospects-A Review. J. Clin. Med..

[144] Jaggi D., Nagamany T., Wolf S., Zinkernagel M.S., Heussen F.M. (2024). Aflibercept for central retinal vein occlusions: Long-term outcomes of a ’Treat-and-Extend’ regimen. BMJ Open Ophthalmol..

[145] Hahad O., Kuntic M., Kuntic I., Daiber A., Munzel T. (2023). Tobacco smoking and vascular biology and function: Evidence from human studies. Pflüg. Arch.-Eur. J. Physiol..

[146] Jimenez M.T.B., Gericke A., Frenis K., Rajlic S., Kvandova M., Kroller-Schon S., Oelze M., Kuntic M., Kuntic I., Mihalikova D. (2023). Effects of aircraft noise cessation on blood pressure, cardio- and cerebrovascular endothelial function, oxidative stress, and inflammation in an experimental animal model. Sci. Total Environ..

[147] Ruan Y., Jiang S., Musayeva A., Gericke A. (2020). Oxidative Stress and Vascular Dysfunction in the Retina: Therapeutic Strategies. Antioxidants.

[148] Deobhakta A., Chang L.K. (2013). Inflammation in retinal vein occlusion. Int. J. Inflamm..

[149] Leavitt J.A., Larson T.A., Hodge D.O., Gullerud R.E. (2011). The incidence of central retinal artery occlusion in Olmsted County, Minnesota. Am. J. Ophthalmol..

[150] Park S.J., Choi N.K., Seo K.H., Park K.H., Woo S.J. (2014). Nationwide incidence of clinically diagnosed central retinal artery occlusion in Korea, 2008 to 2011. Ophthalmology.

[151] Mir T.A., Arham A.Z., Fang W., Alqahtani F., Alkhouli M., Gallo J., Hinkle D.M. (2019). Acute Vascular Ischemic Events in Patients with Central Retinal Artery Occlusion in the United States: A Nationwide Study 2003–2014. Am. J. Ophthalmol..

[152] Schrag M., Youn T., Schindler J., Kirshner H., Greer D. (2015). Intravenous Fibrinolytic Therapy in Central Retinal Artery Occlusion: A Patient-Level Meta-analysis. JAMA Neurol..

[153] Varma D., Lee A.W., Chen C.S. (2014). Reply: ‘A review of central retinal artery occlusion: Clinical presentation and management’. Eye.

[154] Hayreh S.S., Podhajsky P.A., Zimmerman M.B. (2009). Retinal artery occlusion: Associated systemic and ophthalmic abnormalities. Ophthalmology.

[155] Chen C., Singh G., Madike R., Cugati S. (2024). Central retinal artery occlusion: A stroke of the eye. Eye.

[156] Shah R., Zheng X., Patel A.P., Bhatti M.T., Gilbert A., Vora R.A. (2024). Central Retinal Artery Occlusion: Visual Outcomes from a Large Northern California Cohort. Ophthalmol. Retin..

[157] Cheng H.C., Pan R.H., Yeh H.J., Lai K.R., Yen M.Y., Chan C.L., Wang A.G. (2016). Ambient Air Pollution and the Risk of Central Retinal Artery Occlusion. Ophthalmology.

[158] Grzybowski A.E., Mimier M.K. (2019). Evaluation of the Association between the Risk of Central Retinal Artery Occlusion and the Concentration of Environmental Air Pollutants. J. Clin. Med..

[159] Gassel C.J., Andris W., Poli S., Bartz-Schmidt K.U., Dimopoulos S., Wenzel D.A. (2024). Incidence of central retinal artery occlusion peaks in winter season. Front. Neurol..

[160] Srejovic J.V., Muric M.D., Jakovljevic V.L., Srejovic I.M., Sreckovic S.B., Petrovic N.T., Todorovic D.Z., Bolevich S.B., Sarenac Vulovic T.S. (2024). Molecular and Cellular Mechanisms Involved in the Pathophysiology of Retinal Vascular Disease-Interplay Between Inflammation and Oxidative Stress. Int. J. Mol. Sci..

[161] Yokota H., Narayanan S.P., Zhang W., Liu H., Rojas M., Xu Z., Lemtalsi T., Nagaoka T., Yoshida A., Brooks S.E. (2011). Neuroprotection from retinal ischemia/reperfusion injury by NOX2 NADPH oxidase deletion. Investig. Ophthalmol. Vis. Sci..

[162] Cheung S.S.F., Leung J.W.C., Lam A.K.M., Lam K.S.L., Chung S.S.M., Lo A.C.Y., Chung S.K. (2011). Selective over-expression of endothelin-1 in endothelial cells exacerbates inner retinal edema and neuronal death in ischemic retina. PLoS ONE.

[163] D’Amico D.J. (2008). Primary retinal detachment. N. Engl. J. Med..

[164] Ge J.Y., Teo Z.L., Chee M.L., Tham Y.C., Rim T.H., Cheng C.Y., Wong T.Y., Wong E.Y.M., Lee S.Y., SNEC Surgical Retina Research Group (2024). International incidence and temporal trends for rhegmatogenous retinal detachment: A systematic review and meta-analysis. Surv. Ophthalmol..

[165] Popovic M.M., Muni R.H., Kertes P.J., Thiruchelvam D., Chaban Y.V., Qian J., Hillier R., Redelmeier D.A. (2023). A Population-Based Analysis of Long-Term Costs and Adverse Events after Pneumatic Retinopexy and Pars Plana Vitrectomy. Ophthalmol. Retin..

[166] Lin J.B., Narayanan R., Philippakis E., Yonekawa Y., Apte R.S. (2024). Retinal detachment. Nat. Rev. Dis. Primers.

[167] Hazelwood J.E., Mitry D., Singh J., Bennett H.G.B., Khan A.A., Goudie C.R. (2025). The Scottish Retinal Detachment Study: 10-year outcomes after retinal detachment repair. Eye.

[168] Auger N., Rheaume M.A., Bilodeau-Bertrand M., Tang T., Kosatsky T. (2017). Climate and the eye: Case-crossover analysis of retinal detachment after exposure to ambient heat. Environ. Res..

[169] Mansour A.M., Hamam R.N., Sibai T.A., Farah T.I., Mehio-Sibai A., Kanaan M. (2009). Seasonal variation of retinal detachment in Lebanon. Ophthalmic Res..

[170] Laatikainen L., Tolppanen E.M., Harju H. (1985). Epidemiology of rhegmatogenous retinal detachment in a Finnish population. Acta Ophthalmol..

[171] Sevillano Torrado C., Viso E., Moreira S., Blanco M.J., Gude F. (2020). Rhegmatogenous Retinal Detachment and Solar Radiation in Northwestern Spain. Ophthalmologica.

[172] Prabhu P.B., Raju K.V. (2016). Seasonal Variation in the Occurrence of Rhegmatogenous Retinal Detachment. Asia-Pac. J. Ophthalmol..

[173] Lin H.C., Chen C.S., Keller J.J., Ho J.D., Lin C.C., Hu C.C. (2011). Seasonality of retinal detachment incidence and its associations with climate: An 11-year nationwide population-based study. Chronobiol. Int..

[174] Tooulias C., Papaconstantinou D., Droutsas K., Androu A., Theofilou P., Theodossiadis P., Stavrakas P., Georgalas I. (2025). Seasonal Variation of Rhegmatogenous Retinal Detachment: A Systematic Literature Review. Cureus.

[175] Song W., Zhu R., Gao W., Xing C., Yang L. (2022). Blue Light Induces RPE Cell Necroptosis, Which Can Be Inhibited by Minocycline. Front Med..

[176] Cheng K.C., Hsu Y.T., Liu W., Huang H.L., Chen L.Y., He C.X., Sheu S.J., Chen K.J., Lee P.Y., Lin Y.H. (2021). The Role of Oxidative Stress and Autophagy in Blue-Light-Induced Damage to the Retinal Pigment Epithelium in Zebrafish In Vitro and In Vivo. Int. J. Mol. Sci..

[177] Souza Monteiro de Araujo D., Brito R., Pereira-Figueiredo D., Dos Santos-Rodrigues A., De Logu F., Nassini R., Zin A., Calaza K.C. (2022). Retinal Toxicity Induced by Chemical Agents. Int. J. Mol. Sci..

[178] Yu F., Wang Z., Ju B., Wang Y., Wang J., Bai D. (2008). Apoptotic effect of organophosphorus insecticide chlorpyrifos on mouse retina in vivo via oxidative stress and protection of combination of vitamins C and E. Exp. Toxicol. Pathol..

[179] Dong K., Yang N., Ding J., Yan Y.Y., Lu L., Wang Y.S. (2021). RNA interference targeting NOX4 protects visual function in an experimental model of retinal detachment by alleviating blood-retinal barrier damage. Int. J. Ophthalmol..

[180] Ries L., Smith M., Gurney J., Linet M., Tamra T., Young J. (1999). Cancer Incidence and Survival Among Children and Adolescents: United States SEER Program 1975–1995.

[181] Knudson A.G. (1971). Mutation and cancer: Statistical study of retinoblastoma. Proc. Natl. Acad. Sci. USA.

[182] Zhu X.P., Dunn J.M., Phillips R.A., Goddard A.D., Paton K.E., Backer A., Gallic B.L. (1989). Preferential germline mutation of the paternal allele in retinoblastoma. Nature.

[183] Zhu H., Zhao M., Zheng J. (2025). Global, regional, and national burden of retinoblastoma in children aged under 10 years from 1990 to 2021 and projections for future disease burden. Sci. Rep..

[184] Zhou Y., Cai S., Jin M., Jiang C., Xu N., Duan C., Peng X., Zhao J., Ma X. (2020). Economic burden for retinoblastoma patients in China. J. Med. Econ..

[185] Zhang D.L., French D.D., Rossen J.L., Rahmani B. (2023). Direct medical costs of globe salvage in group C-E retinoblastoma and implications for cost-effectiveness. J. Am. Assoc. Pediatr. Ophthalmol. Strabismus.

[186] Berry J.L., Pike S., Rajagopalan A., Reid M.W., Fabian I.D., Afshar A.R., Alejos A., Alemany-Rubio E., Carreras Y.A., Arazi M. (2024). Retinoblastoma Outcomes in the Americas: A Prospective Analysis of 491 Children with Retinoblastoma from 23 American Countries. Am. J. Ophthalmol..

[187] Azary S., Ganguly A., Bunin G.R., Lombardi C., Park A.S., Ritz B., Heck J.E. (2016). Sporadic Retinoblastoma and Parental Smoking and Alcohol Consumption before and after Conception: A Report from the Children’s Oncology Group. PLoS ONE.

[188] Ghosh J.K.C., Heck J.E., Cockburn M., Su J., Jerrett M., Ritz B. (2013). Prenatal exposure to traffic-related air pollution and risk of early childhood cancers. Am. J. Epidemiol..

[189] Heck J.E., Park A.S., Qiu J., Cockburn M., Ritz B. (2015). Retinoblastoma and ambient exposure to air toxics in the perinatal period. J. Expo. Sci. Environ. Epidemiol..

[190] Chen Y., Paul K.C., Walker D.I., Jones D.P., Wang X., Ritz B.R., Heck J.E. (2024). Neonatal per- and polyfluoroalkyl substance exposure in relation to retinoblastoma. Environ. Res..

[191] Murakami T., Yano O., Takahashi H., Akiya S., Higashi K. (1992). Effects of cadmium on the gene expression of retinoblastoma (Y79) cells in culture. Nippon. Ganka Gakkai Zasshi.

[192] Hoek G., van Tongeren M., Röösli M., Jochems S.H.J., Vilahur N., Albin M., Baldi I., Crowley Q., Fervers B., Greinert R. (2026). European Code Against Cancer, 5th edition—Outdoor and indoor air pollution and cancer. Mol. Oncol..

[193] Kim C.H., Kim M.J., Park J., Kim J., Kim J.Y., An M.J., Shin G.S., Lee H.M., Kim J.W. (2021). Bisphenol A Exposure Changes the Transcriptomic and Proteomic Dynamics of Human Retinoblastoma Y79 Cells. Genes.

[194] Garcia H., Villasis-Keever M.A., Zavala-Vargas G., Bravo-Ortiz J.C., Perez-Mendez A., Escamilla-Nunez A. (2024). Global Prevalence and Severity of Retinopathy of Prematurity over the Last Four Decades (1985–2021): A Systematic Review and Meta-Analysis. Arch. Med. Res..

[195] Hong E.H., Shin Y.U., Cho H. (2022). Retinopathy of prematurity: A review of epidemiology and current treatment strategies. Clin. Exp. Pediatr..

[196] Patel A.B., Kumar C.B. (2019). Antenatal exposure to household air pollution and its association with increased risk of retinopathy. Indian J. Child Health.

[197] Li J.H., Zeng H.X., Wei J., Wu Q.Z., Qin S.J., Zeng Q.G., Zhao B., Dong G.H., Shen J.C., Zeng X.W. (2025). Long-term exposure to PM_2.5_ and its constituents and visual impairment in schoolchildren: A population-based survey in Guangdong province, China. Environ. Int..

[198] Brown V.J. (2008). Lead and Retinal Function: Low Prenatal Exposure Suggests Future Abnormality. Environ. Health Perspect..

[199] Polevoy C., Arbuckle T.E., Oulhote Y., Lanphear B.P., Cockell K.A., Muckle G., Saint-Amour D. (2020). Prenatal exposure to legacy contaminants and visual acuity in Canadian infants: A maternal-infant research on environmental chemicals study (MIREC-ID). Environ. Health.

[200] Narotsky M.G., Fuentes L.S., Ola O., Willoughby T.L., Lucas K. (2025). Developmental Toxicity of Disinfection Byproducts in F344 Rats: Effects on Pregnancy Maintenance and Eye Development. Birth Defects Res..

[201] Silver M.K., Shao J., Ji C., Zhu B., Xu L., Li M., Chen M., Xia Y., Kaciroti N., Lozoff B. (2018). Prenatal organophosphate insecticide exposure and infant sensory function. Int. J. Hyg. Environ. Health.

[202] Yang S., Hu H., Kung H., Zou R., Dai Y., Hu Y., Wang T., Lv T., Yu J., Li F. (2023). Organoids: The current status and biomedical applications. MedComm.

[203] Yang C., Du Z., Mei L., Chen X., Liao Y., Ge L., Kang J., Gu Z., Fan X., Xu H. (2025). Influences of lead-based perovskite nanoparticles exposure on early development of human retina. J. Nanobiotechnol..

[204] Gao X., Yuan Y., Lan Y., Lai T., Zhu L., Xu L., Gong J., Ma N., Wang B., Li M. (2025). Polystyrene nanoplastics induced retinal toxicity: Size-, dose-, and developmental stage-dependent effects on human neural retina organoids. J. Hazard. Mater..

[205] Tham Y.C., Li X., Wong T.Y., Quigley H.A., Aung T., Cheng C.Y. (2014). Global prevalence of glaucoma and projections of glaucoma burden through 2040: A systematic review and meta-analysis. Ophthalmology.

[206] Jonas J.B., Aung T., Bourne R.R., Bron A.M., Ritch R., Panda-Jonas S. (2017). Glaucoma. Lancet.

[207] Heijl A., Leske M.C., Bengtsson B., Hyman L., Bengtsson B., Hussein M., Early Manifest Glaucoma Trial Group (2002). Reduction of intraocular pressure and glaucoma progression: Results from the Early Manifest Glaucoma Trial. Arch. Ophthalmol..

[208] Sun Z., Stuart K.V., Luben R.N., Auld A.L., Strouthidis N.G., Khaw P.T., Jayaram H., Khawaja A.P., Foster P.J., UK Biobank Eye and Vision Consortium (2024). Association of Ambient Air Pollution Exposure with Incident Glaucoma: 12-Year Evidence from the UK Biobank Cohort. Investig. Ophthalmol. Vis. Sci..

[209] Huang H.N., Zhu P.P., Yang Z., Tao Y.M., Ma X., Yu H.B., Li L., Ou C.Q. (2024). Joint effects of air pollution and genetic susceptibility on incident primary open-angle glaucoma. Sci. Total Environ..

[210] Chuang S.H., Wu C.L., Le Y.T.T., Chen Y.P., Kuo Y.J., Chang C.H. (2025). Association Between Long-Term Ambient Air Pollution and Primary Open-Angle Glaucoma: A Nationwide Cohort Study in Taiwan. Am. J. Ophthalmol..

[211] Qin D., Yan B., Jiang Y., Guan W., Xu C., Liu Y., Lv J., Wu S., Liu Z. (2026). Ambient air pollution and glaucoma among middle-aged and older adults in China: Evidence from the China health and retirement longitudinal study. Environ. Pollut..

[212] Li Y., Zhu L., Lu M., Wu M., Zhang X., Shi F., Li X. (2026). Short and long-term exposure to fine particulate matter, nitrogen dioxide and meteorological factor and the risk of glaucoma: Evidence from the China health and retirement longitudinal study, distributed lag non-linear and Mendelian randomization models. Int. J. Biometeorol..

[213] Ma Y., Shao M., Li S., Lei Y., Cao W., Sun X. (2024). The association between airborne particulate matter (PM_2.5_) exposure level and primary open-angle glaucoma. Ecotoxicol. Environ. Saf..

[214] Li L., Zhou J., Fan W., Niu L., Song M., Qin B., Sun X., Lei Y. (2021). Lifetime exposure of ambient PM_2.5_ elevates intraocular pressure in young mice. Ecotoxicol. Environ. Saf..

[215] Li L., Xing C., Zhou J., Niu L., Luo B., Song M., Niu J., Ruan Y., Sun X., Lei Y. (2021). Airborne particulate matter (PM_2.5_) triggers ocular hypertension and glaucoma through pyroptosis. Part. Fibre Toxicol..

[216] Li L., Song M., Zhou J., Sun X., Lei Y. (2022). Ambient particulate matter exposure causes visual dysfunction and retinal neuronal degeneration. Ecotoxicol. Environ. Saf..

[217] Lee K., Hong S., Seong G.J., Kim C.Y. (2016). Cigarette Smoke Extract Causes Injury in Primary Retinal Ganglion Cells via Apoptosis and Autophagy. Curr. Eye Res..

